# Dissecting planar and vertical organiser signals in early chick neural development

**DOI:** 10.1242/dev.205685

**Published:** 2026-07-06

**Authors:** Alexandra S. Neaverson, Benjamin J. Steventon

**Affiliations:** Department of Genetics, University of Cambridge, Cambridge CB2 3EH, UK

**Keywords:** Neural induction, Patterning, Node

## Abstract

Early neural development involves a combination of planar signals from the vertebrate organiser and vertical signals from its derived structures, the prechordal plate and notochord, together with early permissive signals from the hypoblast and developing mesendoderm. However, the relative contribution of each organiser-derived structure to neural development is not clear. Here, we isolate anterior tissues from the chick primitive streak at successively later stages of development, to dissect the temporal order of signals from the organiser and its derivatives. Our results show that acquisition of neural identity occurs gradually and that exposure to planar signals from the developing node is necessary for neural plate specification. We also show that planar node-derived signals are required for AP patterning in isolated anterior tissues and give evidence that early neural tissue is of anterior character that subsequently becomes caudalised (in part) by signals from the developing node. However, anterior neural identity is lost without long-term contact with vertical signals from the axial mesendoderm. These results reveal a progressive refinement of neural identity and patterning through a combination of planar and vertical signalling.

## Teaser

**Summary:** Cultures of isolated anterior tissues from the chick embryo reveal the roles of planar and vertical organiser signals for neural specification, and for anteroposterior patterning and maintenance.

## INTRODUCTION

Since the landmark discovery that a graft of the amphibian Spemann-Mangold organiser can induce a fully patterning nervous system composed of host cells, neural development and the organiser have long been intimately linked (Spemann, 1921; Spemann and Mangold, 1924). Equivalent structures were then identified in other organisms, including Hensen's node in birds and mammals, which is located at the top of the primitive streak (Waddington, 1932, 1933, 1934, 1936, 1937; Waddington and Schmidt, 1933; Beddington, 1994; Kinder et al., 2001; Robb and Tam, 2004), with similar inductive abilities. This led to the concept of ‘neural induction’ (NI), in which the neural plate develops in response to instructive signals from the node. As body axis development is accompanied by the self-differentiation of organiser derivatives such as the prechordal plate and notochord, an open question is the degree to which organiser signals operate via planar inductive interactions or through vertical signals from organiser derivatives at later stages of development.

There are multiple processes in early neural development for which planar versus vertical signals may play distinct roles, beginning with the initial specification of the neural plate. In specification assays, explants of the prospective neural plate develop into *SOX2*^+^/*SOX1*^+^ neural (lens-like) tissue in culture (Trevers et al., 2018), demonstrating the degree of autonomous neural development that the epiblast can achieve when isolated from its endogenous signalling environment. Vertical signals from the hypoblast play a role in ‘priming’ the epiblast for further neural development, with FGF signals inducing the expression of pre-neural genes such as *SOX3* and *ERNI* (Streit et al., 2000). Indeed, the developing mesendodermal layer has also been shown to provide permissive vertical signals that are necessary for neural specification to occur (Gallera and Nicolet, 1969; Pera et al., 1999). However, early neural plate explants are sensitive to exogenous epidermal-promoting BMPs until Hamburger and Hamilton stage 4 (HH4), after which point neural development is stable even in the presence of BMPs (Wilson et al., 2000; Wilson and Edlund, 2001). In the context of a node graft, organiser-derived BMP antagonists such as chordin are considered required but not sufficient for the commitment of naïve tissue to neural fate (Streit et al., 1998), since additional organiser-derived signals are required – which are yet to be fully characterised (Lu et al., 2025 preprint). Therefore, continued signalling from the organiser and its derivatives is thought to be essential to fully instruct neural cell fates.

In the chick embryo, *SOX2* expression begins at HH4^+^, around the same time that neural plate specification is complete (Uwanogho et al., 1995; Rex et al., 1997; Linker and Stern, 2004; reviewed by Stern, 2005), and a node graft into the area opaca can induce the expression of *SOX2* (Streit and Stern, 1999; Streit et al., 2000; Linker and Stern, 2004; Trevers et al., 2023). The epiblast layer of the node has been shown to provide planar neural induction signals when grafted into the area opaca (Storey et al., 1995). It has not yet been tested whether the intact neural plate has a continued requirement for organiser-derived signals (including BMP antagonists) to acquire *SOX2* expression. Assessing whether planar organiser-derived signals are required for neural specification requires removing all sources of these signals in the embryo before node derivatives emerge, in addition to other primitive streak regions that are known to contribute to its regeneration (Waddington, 1932; Grabowski, 1956; Yuan et al., 1995a,b; Psychoyos and Stern, 1996; Joubin and Stern, 1999).

Following neural specification, the neural plate becomes subdivided into the prospective forebrain, midbrain, hindbrain and spinal cord territories (Storey et al., 1992; Muhr et al., 1999; Wittler and Kessel, 2004; reviewed by Stern, 2001). A node graft can induce the entire spectrum of AP fates after grafting (Storey et al., 1992; see also Gilbert and Barresi, 2017), but it is not clear the extent to which this depends on early planar signals or later vertical signalling. Studies in *Xenopus* have shown that planar signals from the dorsal mesoderm are key for AP pattern acquisition in the neurectoderm (Altaba, 1992; Doniach et al., 1992) and that AP patterning occurs progressively, with more posterior brain regions passing through a temporary anterior state until they are exposed to later signals from the dorsal mesoderm (Durston et al., 1989; Sive et al., 1989; Cox and Hemmati-Brivanlou, 1995). However, others argue that vertical signals from early involuting mesoderm are the primary signal in *Xenopus* AP patterning (Chen et al., 2000) or that vertical signals are necessary for AP pattern refinement (Poznanski and Keller, 1997). Furthermore, studies in mice emphasise the importance of signals from the anterior axial mesendoderm for patterning and/or maintaining anterior neural tissues (Ang and Rossant, 1993; Camus et al., 2000; Martinez Barbera et al., 2000; Andersson et al., 2006; Fossat et al., 2015; Tam and Masamsetti, 2025).

In the chick embryo, derivatives of the organiser – the axial mesendoderm, which is made up of prechordal mesendoderm, and the notochord – retain the ability to specify defined regions of neural tube along its AP axis (Izpisúa-Belmonte et al., 1993; Foley et al., 1997; Pera and Kessel, 1997; Rowan et al., 1999), suggesting the continued involvement of vertical signals acting from the axial mesendoderm to the overlying neuroepithelium. However, removing the prechordal mesendoderm does not disrupt coarse AP patterning (Pera and Kessel, 1997), with the exception of disrupting the specification of hypothalamic tissue (reviewed by Manning and Placzek, 2024). Prechordal mesendoderm progenitors are located within a defined anterior compartment within the node (Kanno, Rothstein and Simoes-Costa, 2025); it is possible that much of their role in anterior patterning is through planar signalling, and may therefore be complete before they begin their exit from the node at HH4^+^. In addition, signals from non-node sources, such as Wnt, FGF and retinoic acid, are thought to be involved in neural AP pattern establishment (Blumberg et al., 1997; Muhr et al., 1997; Gould et al., 1998; Muhr et al., 1999; Wittler and Kessel, 2004; Nordström et al., 2006). Therefore, it is not clear to what extent vertical signals from axial mesendoderm, planar signals from the organiser and signals from non-axial sources all contribute to the formation and maintenance of the AP pattern.

During axial elongation, axial and paraxial tissues develop in close proximity, so it is possible that they depend on each other for proper morphogenesis, as they do in the tailbud (Xiong et al., 2020). In support of this, grafts of the prechordal plate have been shown to promote convergence of the overlying neuroepithelium (Yoshihi et al., 2022). In contrast, explanted pieces of the neural plate do not show signs of morphogenesis (Muhr et al., 1997; Muhr et al., 1999; Wilson et al., 2000; 2001; Nordström et al., 2006). Yet, *Xenopus* prospective neural plate explants can elongate when sandwiched together with an explant of the chordamesoderm or another neural explant, suggesting that basal contact with another tissue is required for neural plate morphogenesis (Keller and Danilchik, 1988). Therefore, a final unanswered question is whether later stages of neural development can occur in the absence of underlying axial mesendoderm in the chick embryo.

Here, we employ an anterior tissue isolation method to determine the relative contributions of planar signals from the organiser and vertical signals from its derivatives, and the timing that these signals require, for neural specification and anterior-posterior patterning. We find that neural specification is a gradual process that requires planar signals from the anterior primitive streak, and that subsequent planar signals from the node also promote hindbrain specification. Despite anterior neural fates being established early, vertical signals from the underlying axial mesendoderm are important for maintaining forebrain identity. We also find a significant level of autonomy in morphogenesis; once established, the neural plate can form complex structures in the absence of other midline tissues.

## RESULTS

### Planar signals from the developing organiser specify the neural plate

In normal development, *SOX2* is switched on in the early neural plate at HH4^+^ and is generally considered to be the earliest marker of the definitive neural plate (Fig. S4C; see also Uwanogho et al., 1995; Rex et al., 1997; Chapman et al., 2003; Stern, 2005; Sanchez-Arrones et al., 2012). Control of *SOX2* onset is a multi-step process that is not fully understood (Papanayotou et al., 2008). Since the organiser produces a wide range of secreted molecules in the period before *SOX2* expression is initiated, it is possible that this transition requires organiser-derived signals. However, it is also possible that earlier signalling activity from the developing primitive streak establishes commitment to later *SOX2* expression, or that primitive streak-derived signals are simply not required for this transition to occur.

**Fig. 1.**
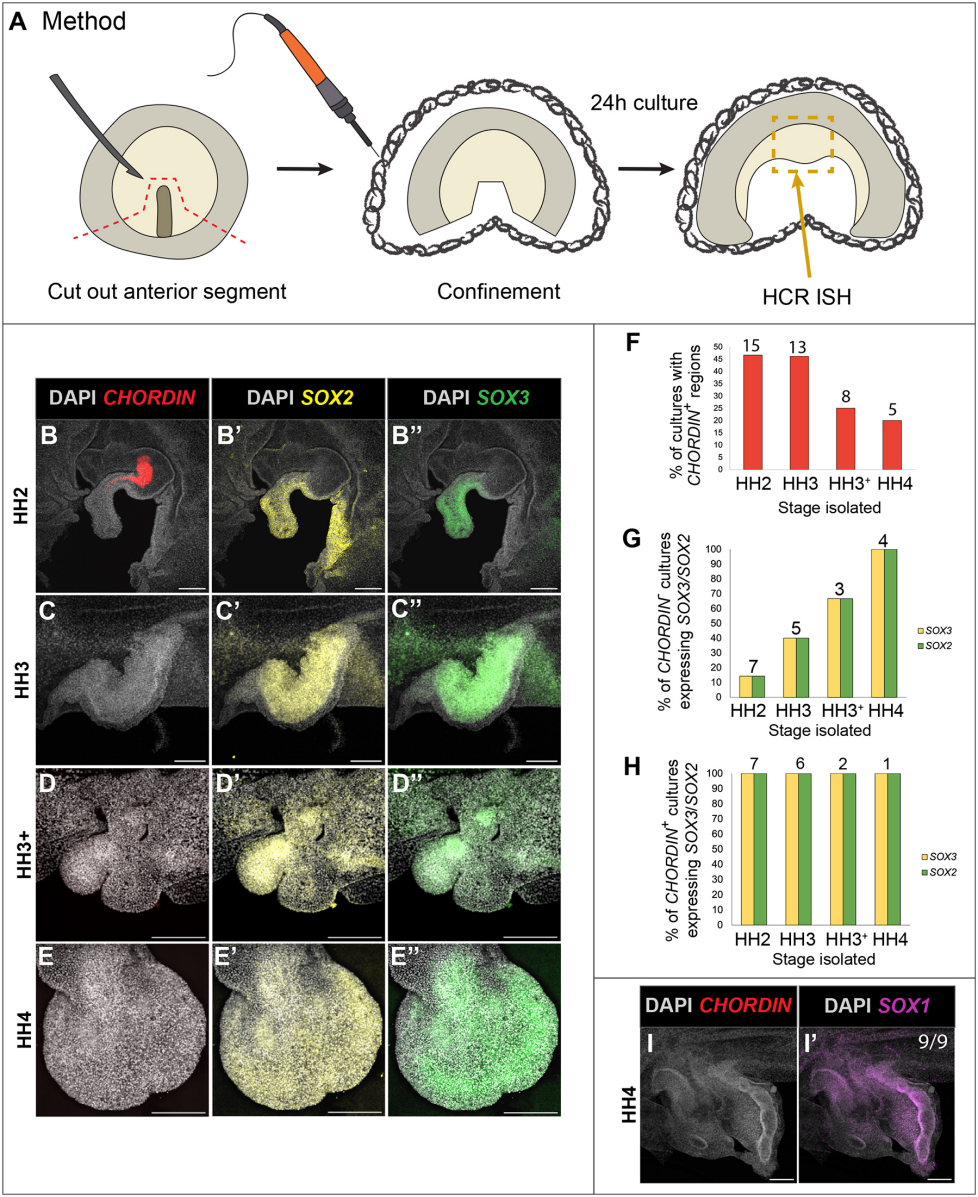
**Neural specification occurs during gastrulation, as the node develops, and is reinforced by the node.** (A) The anterior embryo explant assay was performed by cutting around the node and primitive streak at HH2, HH3, HH3^+^ or HH4. The segment was confined by burning a barrier in the vitelline membrane around the segment using a soldering iron. Segments were cultured for 24 h before HCR FISH staining. (B-E″) Expression of *CHORDIN*, *SOX2* and *SOX3* in the resulting neural tube-like protrusions from anterior segments after culturing for 24 h. (F) Percentage of cultures with *CHORDIN*^+^ regions, indicating node regeneration: 7/15, 6/13, 2/8 and 1/5 cultures had *CHORDIN*^+^ regions at HH2, HH3, HH3^+^ and HH4, respectively. (G) Percentage of cultures that expressed *SOX3* and *SOX2* in the absence of *CHORDIN*: 1/7, 2/5, 2/3 and 4/4 cultures at HH2, HH3, HH3^+^ and HH4, respectively. (H) Percentage of cultures that expressed *SOX3* and *SOX2* that also had *CHORDIN*^+^ regions, showing that the presence of a *CHORDIN*^+^ region guarantees the expression of *SOX3* and *SOX2*: 7/7, 6/6, 2/2 and 1/1 cultures at HH2, HH3, HH3^+^ and HH4, respectively. (I,I′) Example of a HH4 anterior segment 24 h after isolation, stained for *CHORDIN* and *SOX1*. (B-C″,I-I′) 5× maximum intensity projections. Scale bars: 200 μm. (D-E″) 10× maximum intensity projections. Scale bars: 200 μm. Numbers above the bars in F-H indicate sample size.

Previous experiments (Gallera and Nicolet, 1969) and our own data (Fig. S1) indicate that the region of the embryo with organiser ability (i.e. the ability to induce neural gene expression in ectopic locations after grafting) is larger than the node itself. This is reflected in the expression of organiser-associated genes, some of which extend to roughly midway down the primitive streak along its AP axis (Fig. S2; see also Izpisúa-Belmonte et al., 1993; Chapman et al., 2002). In previous node ablation experiments, a much larger area was excised and was followed by complete regeneration (Psychoyos and Stern, 1996; Joubin and Stern, 1999). To study the timing of neural fate establishment in the absence of further node-derived signals, we have used a simple method that we refer to as ‘anterior embryo explant assay’ (Fig. 1A), in which the anterior part of the embryo (containing the prospective neural plate) is isolated from the primitive streak and posterior regions. This is a variation on previous culture techniques (such as those used by Spratt and Haas, 1960; Lipton and Jacobson, 1974; Darnell et al., 1999; Chapman et al., 2003) modified to ensure that all regions of the prospective brain along the AP axis are included (based on early neural fate maps; Fernández-Garre et al., 2002). Since vertical signals from the early non-axial mesendoderm have been shown to play a permissive role in neural specification (Pera et al., 1999), the mesendodermal layer was left intact. Confining the culture is necessary to prevent the epibolic expansion of the extraembryonic tissue. This was achieved using a soldering iron to denature the vitelline membrane, as described by Serrano Nájera et al. (2025) and permitted the assessment of neural development in the absence of the node and primitive streak.

Anterior segments were isolated from embryos at HH2, HH3, HH3^+^, HH4, HH5 and HH6, and cultured for 24 h. During this time, the early neural plate sometimes autonomously develops backward-facing protrusions. The frequency of protrusion formation after isolation at each stage was quantified; isolation after HH4 was associated with an increased ability to form neural tube-like protrusions (Table S1). Hybridisation chain reaction fluorescent *in situ* hybridisation (HCR FISH) was then used to visualise the expression of *CHORDIN*, *SOX2* and the pre-neural marker *SOX3* (Trevers et al., 2018; 2023) (Fig. 1B-E″). An assessment of *CHORDIN* expression in control embryos (Fig. S3) was used to inform the cutting position to minimise the likelihood of leaving behind *CHORDIN*^+^ primitive streak tissue, which may have NI (neural induction) ability.

In this assay, the indicator of neural specification is the ability to express *SOX2* in the absence of *CHORDIN*. Both HH2 and HH3 anterior segments frequently developed *CHORDIN*^+^ regions (Fig. 1B,F), meaning that, at these stages, it is difficult to dissociate neural specification from the potential inductive signals provided by the *CHORDIN*-expressing cells. The appearance of *CHORDIN*^+^ regions indicates the regeneration of node tissue along the cut edge of the culture (similar to observations made by Caldarelli et al., 2024). Node regeneration therefore occurs more frequently in anterior segments from younger embryos.

The incidence of *SOX2* and/or *SOX3* expression without associated *CHORDIN*^+^ tissue increased with isolation at later stages, and we observe a stepwise increase in the frequency of *SOX2/SOX3* expression between HH2 and HH4 (Fig. 1G). In the majority of *SOX2* and/or *SOX3*^+^ cultures, a single expression domain developed, but in two samples (from HH3^+^ and HH4 embryos), two separate domains emerged. This is a potential consequence that excising the primitive streak has on the geometry of the early neural plate. A small number of embryos formed neural tube-like structures and expressed *SOX2* and/or *SOX3* after isolation at HH2-HH3 (Fig. 1C-C″). This suggests that neural specification is a gradual process, rather than a switch occurring at a defined stage, and that the acquisition of neural plate identity is initiated prior to node formation. However, by HH4, it does not require further neural specification signals from the node (100% of cultures express SOX2 after isolation at HH4, Fig. 1G). Therefore, alongside the permissive signals from the underlying mesendoderm, planar signals from the primitive streak provide early neural specification cues. The elongating streak contains precursor cells that will later form the node (Izpisúa-Belmonte et al., 1993; Foley et al., 2000; Streit et al., 2000; reviewed by Stern, 2005). These signals are then superseded by those from the node as it develops, since cultures that were able to regenerate *CHORDIN* tissue (or had *CHORDIN*^+^ left behind) expressed *SOX2* and *SOX3* in 100% of cases (Fig. 1H), suggesting that, although the process is initiated by node precursors, the node provides robustness to the neural specification process.

In later development, *SOX2* is expressed other cell types, such as placodes and neural crest cells, which include cells that do not contribute to the nervous system (Streit, 2002; Roellig et al., 2017). *SOX1* expression begins later in development (HH8, Fig. S4C) and is exclusively expressed in the neural tube (Uchikawa et al., 2011). HH4 anterior segments cultured for 24 h were stained for *SOX1* expression using HCR FISH (Fig. 1I,I′); 9/9 cultures expressed *SOX1* in the absence of *CHORDIN*, demonstrating the progression of neural development in the absence of the node and its derived tissues. This suggests that once neural identity is specified, development can continue without additional planar primitive streak-derived signals.

### Stepwise posteriorisation of the neural plate requires node-derived signals

**Fig. 2.**
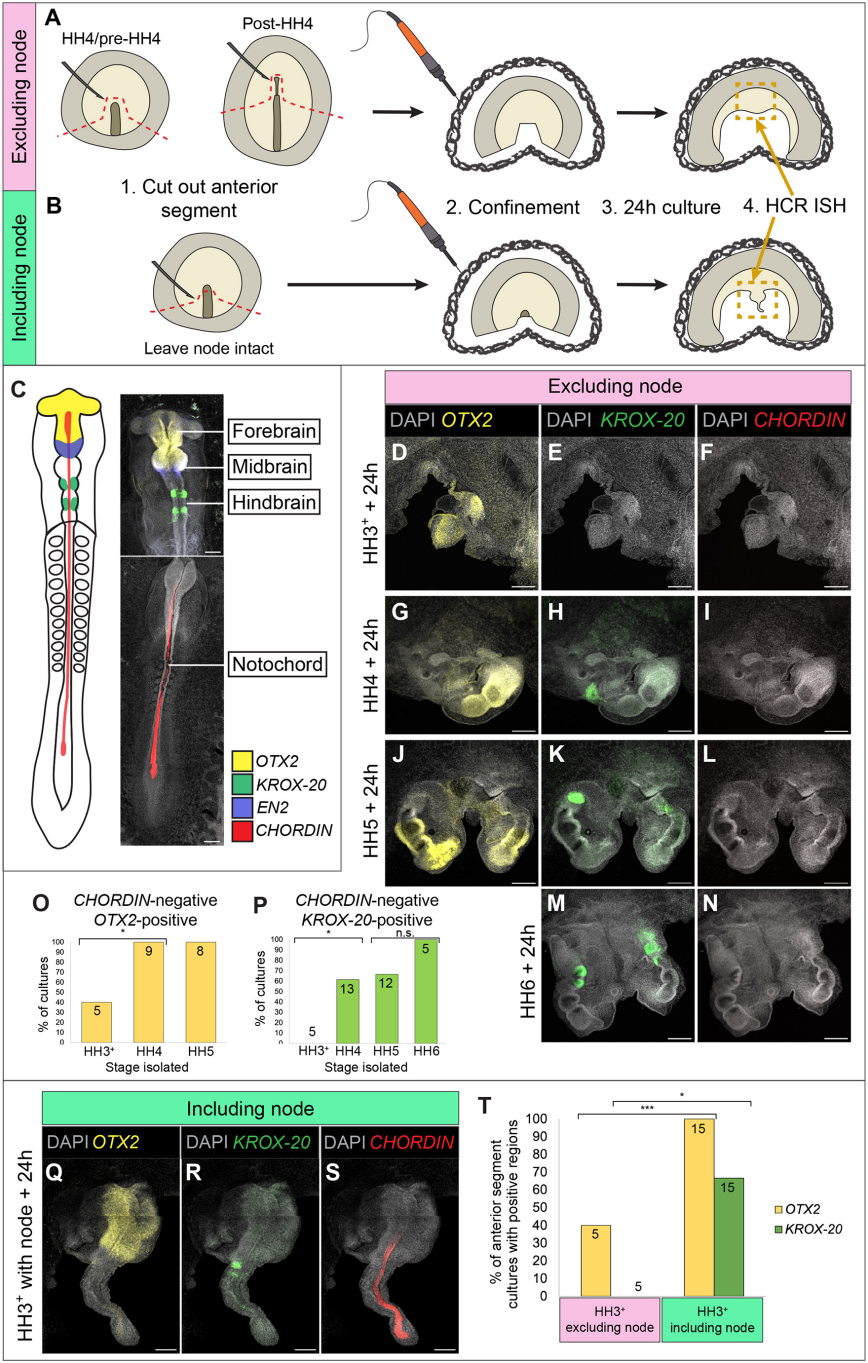
**Anteroposterior patterning of the neural plate through planar signals from the node.** (A) The method of creating and confining the anterior embryo explant assay. The primitive streak and surrounding area were cut away and removed, leaving the neural plate and lateral edges of the embryo with extraembryonic tissue attached, then confined as described in Fig. 1. For stages post-HH4, after the emergence of axial mesendoderm, the cutting was performed around this tissue to exclude it. Anterior segments were cultured for 24 h before HCR FISH staining. (B) The same process as in A was repeated, but the node was left intact. (C) The normal expression pattern for AP patterning genes in the neural tube and *CHORDIN* expression in the notochord, alongside a HCR FISH images. (D-N) DAPI staining and HCR FISH of anterior segments after a 24 h culture showing the expression of forebrain and/or midbrain (*OTX2*), and hindbrain (*KROX20*) marker genes in the absence of node regeneration (*CHORDIN*). (O) Percentage of anterior segment cultures isolated from HH3^+^-HH5 embryos and cultured for 24 h that expressed *OTX2*. Incidence of *OTX2* expression increases between HH3^+^ and HH4 (*P*=0.027). (P) Percentage of anterior segment cultures isolated from HH3^+^-HH6 embryos that expressed *KROX20*. The incidence of *KROX20* expression increases between HH3^+^ and HH4 (*P*=0.036). (Q-S) DAPI staining and HCR FISH of anterior segments isolated at HH3^+^, including the node, after a 24 h culture, showing the expression of *OTX2*, *KROX20* and *CHORDIN*. (T) Percentage of anterior segments isolated at HH3^+^ with or without including the node that expressed *OTX2* (yellow) and *KROX20* (green). 2/5 expressed *OTX2* and 0/5 expressed *KROX20*. With the node included, 15/15 expressed *OTX2* (***P=0.009) and 10/15 expressed *KROX20* (*P=0.033). All statistical analysis was carried out using Fisher's Exact test. Numbers overlaying bars indicate sample size. All images are 5× maximum intensity projections. Scale bars: 200 μm.

We next sought to determine whether vertical signals (i.e. those derived from the axial mesendoderm) are required for AP patterning, or whether node-derived planar organiser signals are sufficient (Fig. 2). The expression of the marker genes used to identify AP patterning is shown in Fig. 2C. *OTX2* is expressed throughout the forebrain and midbrain, while *KROX20* is expressed as four individual domains, which correspond to two bands that appear on both the left and right side of rhombomeres 3 and 5 of the hindbrain. The anterior embryo explant assay was used (Fig. 2A), separating the early neural plate from the primitive streak and posterior body at HH3^+^, HH4, HH5 or HH6. The method was adapted slightly for HH5 and HH6 embryos to cut around the emerging axial mesendoderm. Following a 24 h culture period, anterior segment cultures were HCR-FISH stained for *OTX2*, *KROX20* and *CHORDIN*. At HH3^+^, 40% of cultures expressed *OTX2*, but none expressed *KROX20* (Fig. 2D-F,O,P). This contrasts to cultures isolated at HH4 and HH5, of which 62% and 67%, respectively, expressed *KROX20*, and 100% expressed *OTX2* (Fig. 2G-L,O,P). After isolation at HH6, 100% of cultures expressed *KROX20* in a pattern resembling the rhombomeres of the hindbrain (Fig. 2M,N,P). The number of *OTX2* expression domains was variable, with all cultures having between 2 and 4 separate *OTX2*^+^ regions; this was independent of the stage isolated. In contrast, the mean number of *KROX20^+^* domains increased with isolation stage [HH4–0.92 domains (*n*=12); HH5–1.42 domains (*n*=12); HH6–2.2 domains (*n*=5)]. Since 62% of HH4 cultures were able to express *KROX20* following 24 h culture, this indicates that AP pattern acquisition, at least up to hindbrain level, primarily occurs through cues from planar, node-derived signals between HH3^+^ and HH4. This supports the idea that the first neural tissue to develop is of anterior character (‘Activation’, as shown by the *OTX2*^+^ cultures isolated at HH3^+^), of which some parts are then transformed into more posterior regions (‘Transformation’) (Nieuwkoop, 1952).

The reduced expression of *KROX20* relative to *OTX2* after isolation at HH4-HH5 could indicate that posteriorisation of the neural plate is influenced by patterning signals for a longer duration than more-anterior regions. These cues may be planar signals from the node, vertical signals from the later-forming axial mesendoderm or planar signals from other tissues with known patterning properties – such as the posterior streak and/or epiblast and paraxial mesoderm (reviewed in Wilson and Houart, 2004) that are absent from the culture. To separate these possibilities, the anterior embryo explant assay was repeated at HH3^+^ with the node left intact (Fig. 2B). 100% of these cultures expressed *OTX2*, while 60% expressed *KROX20* (Fig. 2Q-T). However, 100% of cultures isolated at HH6 without the node and/or axial tissues expressed *KROX20* (Fig. 2P). This suggests that signals from axial tissues are sufficient and required for the stabilisation of *OTX2* expression. Non-axial tissues providing caudalising signals (like the emerging paraxial mesoderm) likely act in conjunction with the node between HH4 and HH6 to stabilise *KROX20* pattern. Overall, this indicates that AP patterning occurs in a stepwise fashion and is predominantly orchestrated by planar node-derived signals, but hindbrain fates take longer to fully establish, and may be supported by additional caudalising signals from axial and non-axial mesoderm.

### Neural morphogenesis can occur without node-derived axial mesendoderm

Previous studies have indicated that the anterior axial tissues – the head process and prechordal plate – interact with the overlying neural plate during head formation (Yoshihi et al., 2022). Yet, the anterior segment cultures produce extensive neural tube-like structures, especially when isolated at HH4 or later (Figs 1 and 2), showcasing the intrinsic ability of the neural plate to fold up and undergo morphogenesis in the absence of the mesendodermal and paraxial tissues (Moury and Schoenwolf, 1995; Schoenwolf and Yuan, 1995; Smith and Schoenwolf, 1997; Lawson et al., 2001).

**Fig. 3.**
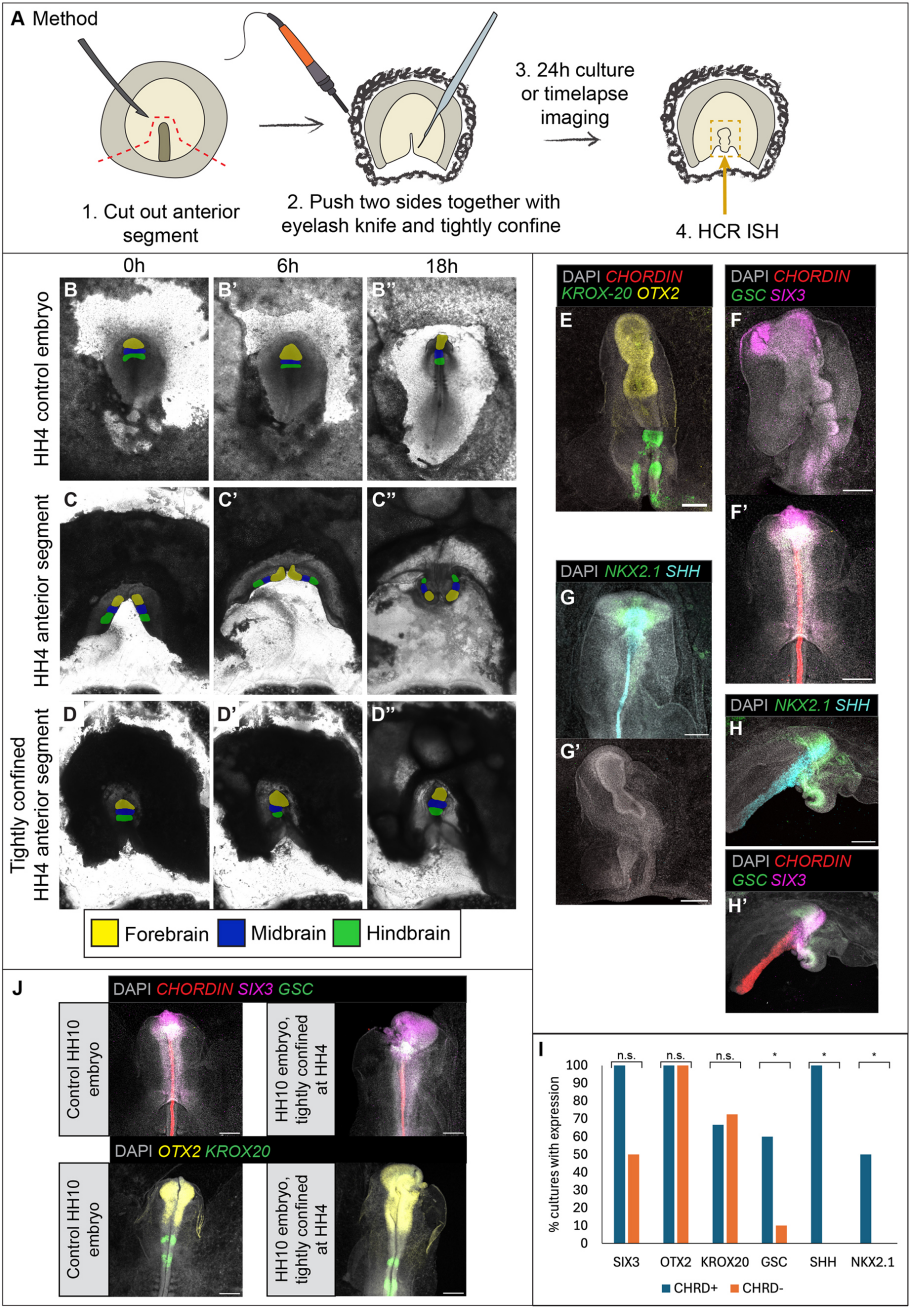
**Anterior segment cultures become inverted without midline tissue, but can be rescued by tight confinement.** (A) The method of tightly confining the anterior embryo explant assay. Method is as described in Fig. 2A, but step 2 additionally involves pushing the two lateral pieces of tissue together with an eyelash knife, and burning the barrier in the vitelline membrane more closely to the tissue. (B-D″) Stills from time-lapse movies showing the development of (B-B″) a control embryo, (C-C″) a HH4 anterior segment and (D-D″) a tightly confined anterior segment, during an 18 h culture. Masks were manually added to show the approximate position of the forebrain (yellow), midbrain (blue) and hindbrain (green). (E,F) Tightly confined HH4 anterior segments after HCR FISH to demonstrate the correct relative positions of (E) *OTX2* and *KROX20*, without *CHORDIN* present, and (F) *SIX3*, without *GSC* and *CHORDIN* present. (F′) Control HH10 embryo with HCR FISH staining for *CHORDIN*, *GSC* and *SIX3*. (G) Control HH10 embryo with staining for *NKX2.1* and *SHH*. (G′) Tightly confined HH4 anterior segment with staining for *NKX2.1* and *SHH*. (H,H′) A tightly confined HH4 anterior segment demonstrating that *CHORDIN* expression is associated with the co-expression of *NKX2.1*, *SHH*, *GSC* and *SIX3*. (I) Quantification of the percentage of anterior segments (including both tightly and loosely confined samples) expressing markers of neural AP pattern (*SIX3*, *OTX2* and *KROX20*), prechordal plate (*GSC*), floorplate and/or notochord (*SHH*), and ventral forebrain (*NKX2.1*), in the presence of absence of *CHORDIN*^+^ tissue. The presence of *GSC* (*P*=0.042), *SHH* (*P*=0.012) and *NKX2.1* (*P*=0.039) is associated with the co-expression of *CHORDIN*. Total number of samples for each gene are as follows. For *CHRD*^−^ samples, *SIX3 n*=20, *OTX2 n*=25, *KROX20 n*=29, *GSC n*=20, *SHH n*=6, *NKX2.1 n*=14. For *CHRD*^+^ samples, *SIX3 n*=5, *OTX2 n*=4, *KROX-20 n*=6, *GSC n*=5, *SHH n*=3, *NKX2.1 n*=4. (J) HH10 control embryo from F′ (left) compared to a HH10 embryo tightly confined from HH4 (right) to demonstrate that tight confinement does not modify the overall localisation of expression for any of the genes tested. All statistical analysis was carried out using Fisher's Exact test. All images are 5× maximum intensity projections. Scale bars: 200 μm.

The orientation of marker gene expression in the neural tube-like structures is often inverted relative to normal; for example, in Fig. 2G-L, the expression of *OTX2* appears in a more posterior position than that of *KROX20*. To better understand this apparent axis inversion, a timelapse recording of an anterior segment culture from a HH4 embryos was obtained; stills are shown in Fig. 3C-C″ (Movie 2). Masks over the forebrain, midbrain and hindbrain regions were manually added to demonstrate the movement of the tissues over time, compared to an unoperated control embryo (Fig. 3B-B″, Movie 1). In the absence of the midline, the early neural plate is split instead of developing as one coherent structure. The two halves develop and form complex structures independently (as described by Darnell, Schoenwolf and Ordahl, 1992), including inflated brain vesicles that then push anteriorly as they grow and collide with the opposite side. As they continue to grow, the two sides push against each other and end up protruding backwards into the gap (Fig. 3C″), resulting in the development of an inverted inside-out brain-like structure.

To understand whether this purely results from the altered geometry after removing the midline tissue, the anterior segment culture method was modified slightly (Fig. 3A): the two sides of the culture were forced into contact at the midline, then the culture was tightly confined to prevent expansion of the tissue. Healing occurred and the neural plate developed in this position rather than becoming inverted (Fig. 3D-D″, Movie 3). AP markers of the brain – *OTX2*, *KROX20* and the anterior forebrain marker *SIX3* – were expressed in the correct orientation and position (Fig. 3E,F; for *SIX3*, compare to normal expression in Fig. 3F′), and the tissues developed into something that more closely resembled the normal brain. Tight boundaries for *OTX2* and *KROX20* expression are observed (Fig. 3E). These resemble the normal expression pattern (Fig. 2C) more closely than the previous loosely confined cultures. Tight confinement itself was not found to impact the overall expression pattern for AP and axial mesendoderm markers compared to controls, when tested on whole embryos (Fig. 3J). The frequencies of *SIX3*, *OTX2* and *KROX20* expression with and without *CHORDIN* present were compared, and no significant difference was found (Fig. 3I). Overall, this shows that the AP orientation of the neural tube is stable once patterning has occurred (at HH4) so further signals from the axial mesendoderm do not substantially impact this AP pattern.

The AP patterning displayed in these cultures prompted us to ask whether they also showed signs of DV patterning. The axial tissues, including the prechordal plate, have a well-known role in specifying the DV axis of the brain by producing a graded SHH signal (Pera and Kessel, 1997; Briscoe and Ericson, 2001). However, SHH is produced by the node prior to the formation of the prechordal plate and notochord (Patten et al., 2003; Levine and Brivanlou, 2007). Furthermore, some evidence suggests that, in the chick, specification of the floorplate – the ventral-most part of the neural tube – precedes the expression of SHH in the notochord (Kremnyov et al., 2018). Therefore, the first steps of DV patterning may begin prior to axial mesendoderm formation, through planar interactions. On that note, we looked for the expression of the floorplate and/or notochord marker *SHH*, the prechordal plate marker *GSC* and the ventral forebrain and/or hypothalamic marker *NKX2.1* using HCR FISH in anterior segments isolated at HH4. *SHH* and *NKX2.1* expression were rarely observed (Fig. 3G,G′); the few cultures expressing *NKX2.1* and *SHH* were also found to co-express *CHORDIN* (Fig. 3H,H′), indicating that no ventral brain specification was observed in the absence of notochord tissue. In addition, the expression of *GSC* was also associated with the presence of *CHORDIN*, suggesting that prechordal plate tissue tends to be present in cultures with node regeneration or leftover node tissue. Signs of DV patterning only appear when *CHORDIN* is present, indicating that vertical signals from the axial mesendoderm are required for DV patterning, but not for AP patterning (Fig. 3I).

### Long-term maintenance of forebrain identity requires node-derived axial mesendoderm

Anterior identity of neural tissues is established at or prior to HH3^+^, but studies in other vertebrate embryos, in particular the mouse, indicate that vertical signals from node-derived axial mesendoderm (e.g. the prechordal plate) are important for maintaining forebrain identity (Martinez Barbera et al., 2000; Andersson et al., 2006). If this holds true for the chick embryo, a decline in forebrain marker expression might occur following prolonged culture without node-derived tissues. Since *OTX2* is expressed in both the forebrain and midbrain, we additionally stained the cultures for *SIX3*, a forebrain-specific marker that initiates its expression at HH4 (Fig. S4B).

**Fig. 4.**
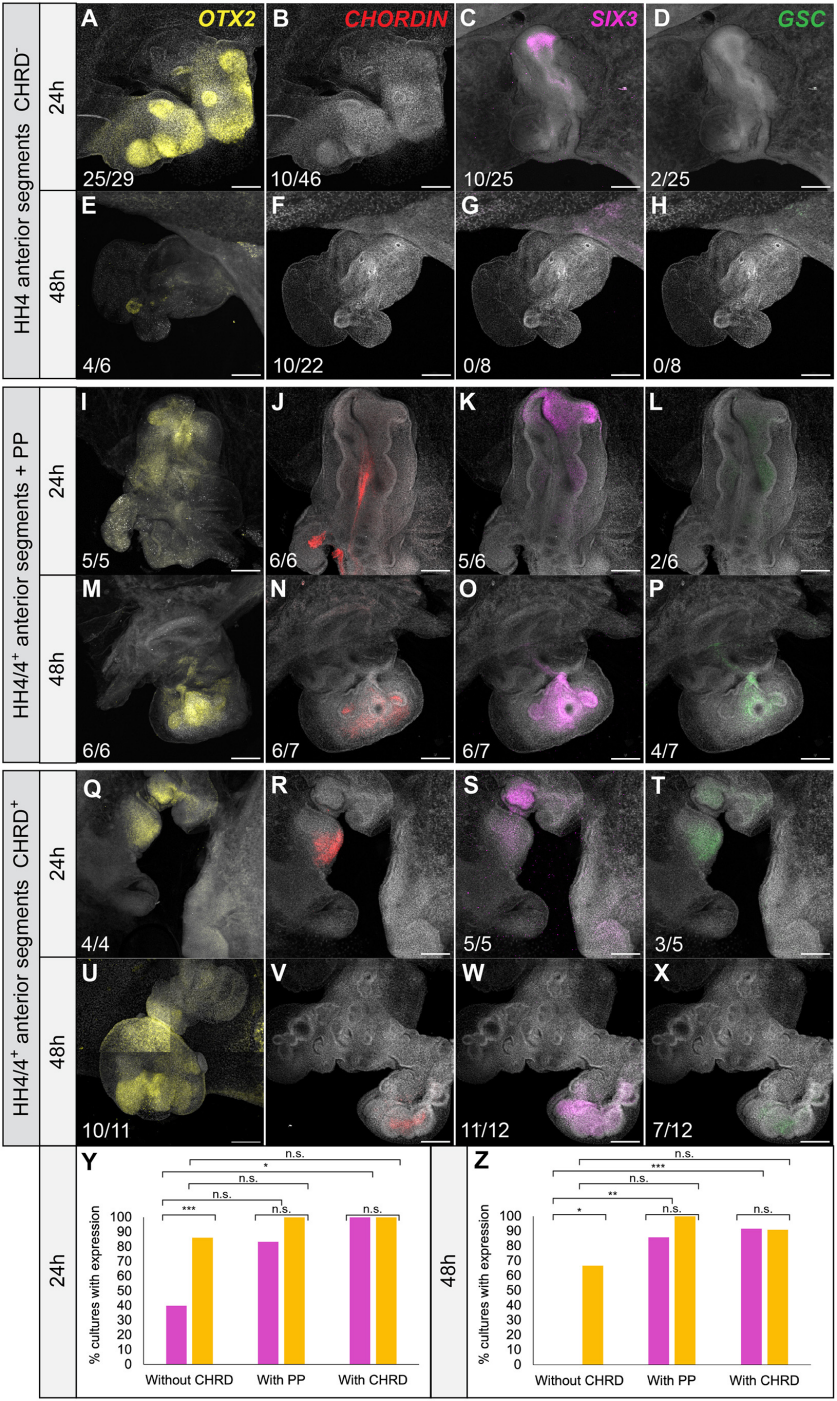
**Loss of forebrain identity in the absence of the node and its derivatives.** (A-H) Anterior segments were isolated from the primitive streak and posterior part of the embryo at HH4, as previously described, and cultured for 24 h (A-D) or 48 h (E-H) before HCR FISH staining for *OTX2*, *CHORDIN*, *SIX3* and *GSC*. Images and quantification are for samples that did not express *CHORDIN*. Note that for the scoring of expression, the *CHORDIN*^−^ 24 h samples without prechordal plate (PP) (A-D) include samples from both tight and normal confinement, while all other samples underwent normal confinement. (I-P) Anterior segments were isolated from the primitive streak and posterior part of the embryo at HH4-HH4^+^, but with prechordal plate (PP) tissue left intact, then cultured for 24 h (I-L) or 48 h (M-P) before HCR FISH staining. (Q-X) Anterior segments were isolated from the primitive streak at HH4-HH4^+^, as previously described, and cultured for 24 h (Q-T) or 48 h (U-X) before HCR FISH staining. Images and quantification are for samples that did express *CHORDIN*. Scores indicate the number of samples positive for any given gene. All images are 5× maximum intensity projections. Scale bars: 200 μm. (Y,Z) Quantification of the percentage of anterior segments at 24 h (Y) or 48 h (Z) with expression of *OTX2* (orange) and *SIX3* (magenta). At 24 h (Y), without *CHORDIN* present, *OTX2* is expressed in significantly more samples than *SIX3* (***P=0.0005), and *CHORDIN* is associated with co-expression of *SIX3* (*P=0.042). At 48 h (Z), *SIX3* is not expressed in any samples without *CHORDIN*, while *OTX2* is expressed in 67% of samples (*P=0.015). The presence of the prechordal plate (PP) (**P=0.0014) or *CHORDIN* expression (***P=0.00007) are associated with the expression of *SIX3* at 48 h.

Anterior segments isolated at HH4 were cultured for 48 h, before HCR staining for *OTX2* or *SIX3*, and compared against anterior segments cultured for 24 h (Fig. 4). As previously described, after 24 h culture, HH4 anterior segments consistently express *OTX2* (Fig. 4A). At 48 h, 4/6 *CHORDIN*-negative cultures expressed *OTX2*, but the fluorescence intensity was markedly reduced (Fig. 4E). Most *CHORDIN*-negative cultures did not express *GSC* at 24 h (Fig. 4D) and none expressed *GSC* at 48 h (Fig. 4H), indicating that the prechordal plate was absent. For *SIX3*, expression at 24 h appeared less frequently than *OTX2* – 10/25 cultures lacking *CHORDIN* expressed *SIX3* (Fig. 4C). At 48 h, 0/8 *CHORDIN*-negative cultures expressed *SIX3* (Fig. 4G). Forebrain identity is therefore sensitive to signals from the axial mesendoderm when cultured for a prolonged period.

To test whether the reduced expression of *SIX3* could be rescued by the prechordal plate, the cultures were repeated at HH4-HH4^+^ with this tissue left intact (Fig. 3I-P). At HH4^+^, the prechordal plate can be seen as the first cells begin to migrate anteriorly out of the node, while at HH4, these cells are still contained within the node in its anterior-most quadrant (Kanno, Rothstein and Simoes-Costa, 2025). We could visualise *GSC* expression in only 2/6 cultures at 24 h, and 4/7 cultures at 48 h, likely due to a combination of weak expression, rare cell population and depth of the cells within the sample. However, the presence of axial mesendoderm was confirmed by the presence of *CHORDIN* (Fig. 3J,N). Prechordal tissues did not significantly impact the expression of *SIX3* or *OTX2* at 24 h, but boosted the expression of *SIX3* at 48 h (Fig. 4O,Y,Z).

Several cultures included *CHORDIN*^+^ tissue, either as a result of node regeneration or incomplete removal of node tissues. All cultures with visible *CHORDIN* (including those with the prechordal plate included) were grouped and compared to those without *CHORDIN* (Fig. 4Q-Z). At 24 h, the presence of *CHORDIN* was associated with the presence of *SIX3*. By 48 h, this association strengthens, as *SIX3* was not found in any cultures lacking *CHORDIN*. No significant association was found for *OTX2*.

Overall, this suggests that prolonged contact with signals from the underlying axial mesendoderm is important for long-term expression of forebrain-specific genes. This is particularly apparent for forebrain-specific *SIX3*, while *OTX2* – which could indicate forebrain or midbrain tissue – is retained but appears to be only weakly expressed. Since isolation of these anterior cultures was performed at HH4-HH4^+^ (when *SIX3* can be first detected in the anterior neural plate), from these findings we cannot rule out the possibility that some cultures may have been isolated before the onset of *SIX3* expression. These findings suggest a late role for the node, or a role for its early axial derivatives, in the initial induction and/or maintenance of *SIX3* expression.

## DISCUSSION

**Fig. 5.**
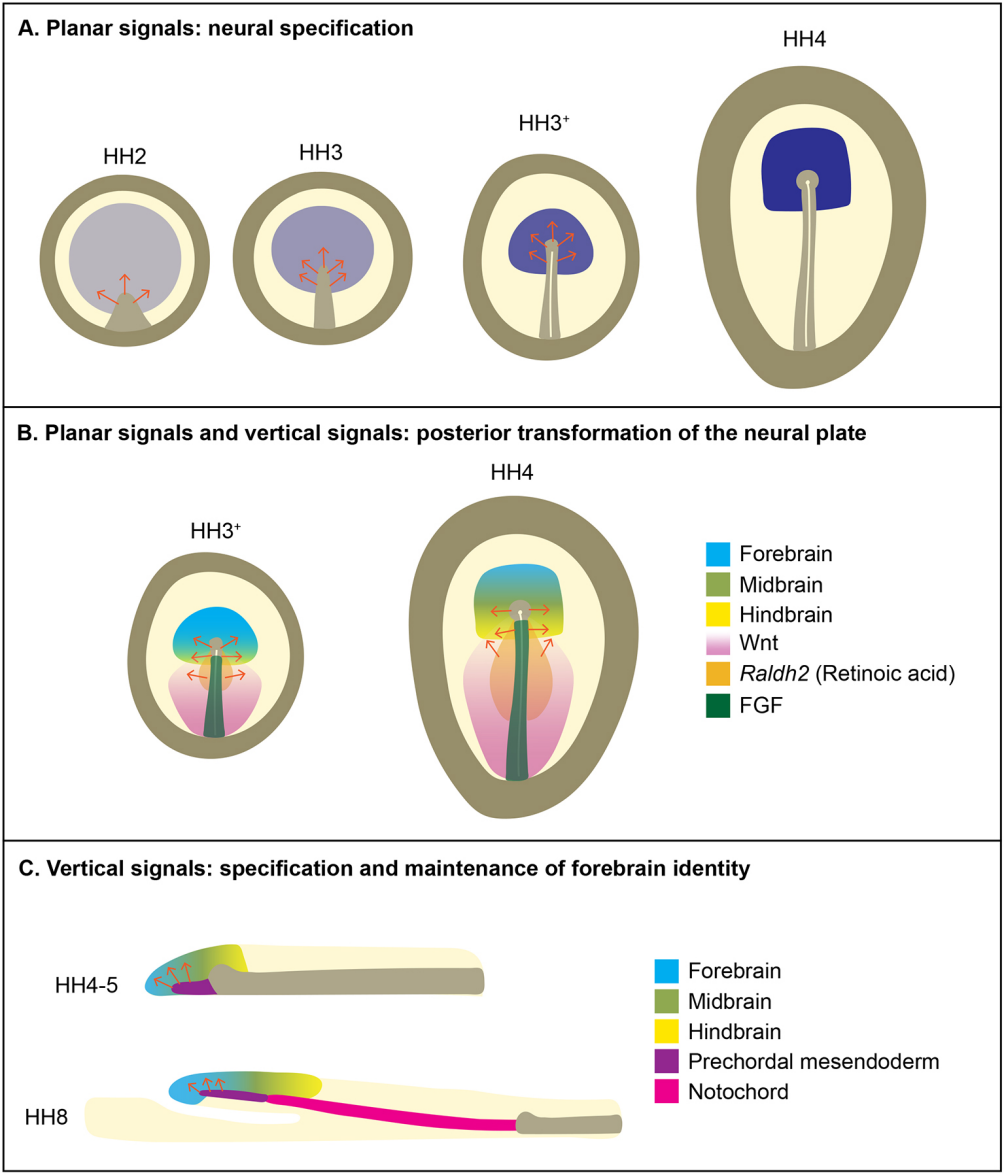
**Summary of the contribution of planar and vertical signals to neural specification, AP regionalisation and forebrain patterning in early chick development**. (A) Planar signals act to specify the neural plate. By HH2, the epiblast exists in a pre-neural forebrain-like state, primed for neural specification. During HH2-HH3, the primitive streak develops, providing signals (arrows) that pass along the plane of the epiblast to specify the neural plate. This is a gradual process that occurs concomitantly with primitive streak and node development. It continues through HH3^+^, when the neural specification signals become concentrated in the anterior half of the primitive streak, including in the developing node. By HH4-HH4^+^, the neural plate is now specified and *SOX2* expression begins. (B) Planar and vertical signals (arrows) contribute to the posterior transformation of the neural plate. As the neural plate is becoming specified, it also becomes regionalised along its AP axis. The node provides planar signals that act to transform adjacent parts of the neural plate (previously of anterior character) into posterior neural tissue, specifying the midbrain and hindbrain by HH4. Planar posteriorising signals are also provided by the primitive streak (FGF) (Storey et al., 1998) and the posterior epiblast (Wnt) (Muhr et al., 1997; Muhr et al., 1999; Nordström, Jessell and Edlund, 2002; Nordström et al., 2006). At the same time, paraxial mesoderm progenitors ingress through the primitive streak and start to emerge beneath the epiblast, expressing *RALDH2* to provide retinoic acid signals that also contribute to the posterior transformation of the neural plate (Muhr et al., 1999). (C) Vertical signals (arrows) contribute to the specification and maintenance of forebrain identity. At HH4-HH5, prechordal mesendoderm (including prechordal plate) cells migrate anteriorly out of the node, providing signals that act vertically on the overlying neural tissue to specify the anterior and/or ventral forebrain. During the next 24 h of development, the prechordal mesendoderm and notochord have become resolved and continue to provide vertical signals that set up the DV axis of the neural tube, and the prechordal mesendoderm continues to signal to maintain and refine neural identities in the anterior and/or ventral forebrain.

In this work, we separate the involvement of planar primitive streak-derived signals from vertical axial mesendoderm-derived signals during early chick development, providing a timeline for the involvement of the developing organiser, the node proper, and the axial mesendoderm for the development and maintenance of neural tissues in the chick embryo. We propose a model for the involvement of these tissues in Fig. 5.

The anterior embryo explant assay permits the separation of the neural plate from sources of NI signals – allowing us to exclude the entire primitive streak – at defined stages. In doing this, we show that, rather than a simple switch, neural fate is gradually established through planar signals from the primitive streak during node development. Small neural plate explants from HH3 embryos develop into *SOX2*^+^ neural tissue in culture, but upon exposure to BMP signals develop into epidermis (Muhr et al., 1999; Wilson and Edlund, 2001). Then, at HH4, a change occurs that allows neural plate explants to develop into neural tissue, even in the presence of BMP. We show that our cultures, which can be thought of as whole neural plate explants, rely on organiser-derived signals between HH2 and HH4 to develop *SOX2*^+^ neural tissue. Our experiments demonstrate that a neural plate without an intact organiser will not initiate *SOX2* expression, and that *SOX3* expression – although initiated before primitive streak development (Rex et al., 1997) – drops unless neural specification has occurred, since *SOX3* is never observed in the absence of *SOX2*. This fits with the observations from organiser graft experiments showing that the initial ‘pre-neural’ state of the epiblast, induced by FGF, is unstable unless exposed to further signals from the organiser (Streit et al., 2000). Our results show that signals from the organiser (defined as the anterior half of the primitive streak at HH2-HH4) are required for *SOX2* expression and neural specification during normal development. These signals likely work in conjunction with permissive signals from the developing mesendoderm (Pera et al., 1999).

Anterior segments separated from organiser-derived signals at HH4 continue to develop and initiate expression of *SOX1* (Fig. 1I,I′), a pan-neural gene that is not expressed until HH8 (Fig. S4D). This suggests that, once the neural plate is specified, organiser-derived signals are no longer required, and neural development can progress autonomously. This may be the result of an auto/cross-regulatory loop involving the SOXB1 genes (which include *SOX1*, *SOX2* and *SOX3*), since ectopic expression of *SOX2* has been shown to cause *SOX1* expression (Graham et al., 2003). Therefore, *SOX2* may directly initiate *SOX1* expression.

The anterior embryo explant assays are similar to the rostral blastoderm isolates performed by Darnell et al. (1999) and Chapman et al. (2003). They found that neural specification occurs by stage 3d (which corresponds to between HH3^+^ and HH4), which broadly fits with our timeline. A key distinction between our work and previous experiments is the use of multiplex HCR to simultaneously stain for organiser genes and neural plate markers. This was important, since we frequently detected regions of *CHORDIN* expression indicating node regeneration. It allowed us to conclude that the presence of *SOX3* and/or *SOX2* in cultures isolated at early stages (e.g. HH2) is often correlated with the presence of a regenerated node, and to therefore exclude these samples from quantification. Furthermore, the anterior segments were grown in EC culture [as opposed to using Spratt's technique (Spratt, 1947)], which likely permits morphogenesis in a way that more closely resembles normal development, since the tissues remain in contact with the vitelline membrane. Our anterior segment cultures are somewhat similar to the classical ‘animal cap assays’ performed in *Xenopus*, which involve culturing the animal cap, previously exposed to early neuralising signals (Stern, 2005) in isolation, or in the presence of other tissues or defined signalling molecules (Green, 1999). However, our cultures include extraembryonic tissue, a known source of anti-neural BMP signals (Streit et al., 1998). This allowed us to test the requirement of organiser-derived signals in a system that more closely resembles the normal signalling environment of the developing neural plate.

Previous research across vertebrate models broadly demonstrates that AP patterning can occur in the absence of vertical signalling from the underlying organiser-derived mesendoderm (Doniach et al., 1992; Pera and Kessel, 1997; Schier et al., 1997; Strähle et al., 1997; Feldman et al., 1998; Fossat et al., 2015; Kakebeen et al., 2021; Manning and Placzek, 2024). However, prechordal plate removal experiments do not exclude the possibility that AP patterning signals are produced by these cells as they migrate out of the node. To exclude this possibility, experiments that completely prevent the formation of anterior axial mesendoderm are needed; this is achieved when the anterior embryo explant assay is performed in embryos at HH4 or younger. The results of our anterior segment experiments stained for *OTX2* and *KROX20* (Fig. 2) are concordant with previous research: defined forebrain and/or midbrain, and hindbrain tissues emerge within 24 h, after isolation from axial mesendoderm before any vertical signalling from these tissues could have occurred. Our experiments additionally reveal differences in the timing of signal requirement for the anterior neural tissues compared to the more-posterior neural tissues. The presence of *OTX2* without *KROX20* in HH3^+^ cultures, combined with the increase in *KROX20* expression up to HH6, suggests that genes expressed in the early embryo that later become restricted to the anterior – such as *OTX2* – become committed before genes expressed in more posterior neural tissues, such as *KROX20*. These results give evidence for Nieuwkoop's activation-transformation model of neural development (Nieuwkoop, 1952) and resemble those of the experiments carried out by Sive et al (1989) in *Xenopus*, who demonstrated that cells initially specified as the far-anterior cement gland tissue are transformed into more posterior fates after prolonged contact with dorsal mesoderm. Further work is needed in order to establish whether the *KROX20*-expressing hindbrain emerges from a previously *OTX2*^+^ population or a separate group of cells.

The increasing incidence and number of domains of *KROX20* expression up to HH6 suggests that additional signals and/or time are required to specify the hindbrain compared to the fore- and/or midbrain. The lack of sharp boundaries of *KROX20* expression, and the reduced number of *KROX20*^+^ domains in cultures isolated prior to HH6 could reflect insufficient contact with hindbrain-promoting Wnt signals before isolation, or contributions from the underlying axial mesoderm to refining AP pattern (Rowan et al., 1999). The paraxial mesoderm is an additional source of signals, including Wnt, that is known to promote posterior neural fates in chick, zebrafish and *Xenopus* (Muhr et al., 1997; Woo and Fraser, 1997; Muhr et al., 1999; Elkouby et al., 2010). Prolonged contact with these signals post-HH4 may be the reason why *KROX20* domains appear more refined and more frequently in cultures isolated at HH5-HH6.

Overall, our findings on AP patterning align with previous experiments using neural plate explants, showing the stepwise posteriorisation of the neural plate over time (Muhr et al., 1997; Muhr et al., 1999; Nordström, Jessell and Edlund, 2002; Nordström et al., 2006), and show that their findings extend to a whole-neural plate context. It remains to be seen whether spinal cord specification occurs in these cultures; there is ongoing debate about whether any part of the spinal cord arises through activation-transformation (Nieuwkoop, 1952) or from bipotent neuromesodermal progenitors (NMps) (Cambray and Wilson, 2002, 2007; Tzouanacou et al., 2009; Wymeersch et al., 2016). Such an experiment would require precise co-staining for neural and mesodermal markers, alongside lineage analysis, in order to rule out the presence of NMps.

In Fig. 3, we demonstrate that early brain orientation and morphogenesis does not depend on the presence of underlying axial mesendoderm. The continued morphogenesis is consistent with experiments showing that neural tube elongation continues when notochord extension is stalled (Charrier et al., 1999) or when the notochord is absent in mouse mutants (Ang and Rossant, 1993). These results are similar to those of previous tissue isolation experiments (Smith and Schoenwolf, 1991; Moury and Schoenwolf, 1995; Schoenwolf and Yuan, 1995; Lawson et al., 2001), further confirming that morphogenetic forces in anterior neural tissues are tissue intrinsic.

Despite the strong resemblance to normal brain development, we show that anterior segments isolated at HH4 do not have any signs of patterning along the DV axis, as shown by the lack of *SHH* and *NKX2.1* expression (Fig. 3G′). The only cultures expressing these genes were those that also expressed *CHORDIN*, indicating the presence of notochord tissue, which has a known role in DV patterning (Placzek et al., 1990; Yamada et al., 1991). A gradient of secreted SHH from the notochord and floorplate is the primary driver of DV patterning (Echelard et al., 1993; Roelink et al., 1994; Ericson et al., 1995, 1996). Although SHH is expressed in the node prior to the extension of the axial mesendoderm, our results suggest that DV patterning of the neural tube does not occur until after these mesendodermal tissues come to underlie the developing neural tube. In contrast to AP patterning, which is well under way by HH4, DV patterning occurs later through vertical signalling.

Finally, extended culture of anterior segments revealed that genes associated with forebrain/midbrain identity are not maintained in the absence of axial mesendoderm (Fig. 4). The fading expression of *OTX2* at 48 h (Fig. 4E) resembles effects seen when the early emerging prechordal mesendoderm is removed at HH5 (Pera and Kessel, 1997). The effect on *SIX3* was more pronounced, with fewer than half of the cultures expressing *SIX3* at 24 h (Fig. 4C). Since *SIX3* expression begins at HH4 (Fig. S4B), this can be interpreted in two ways: either its expression has already begun to decline at 24 h, or the isolation was performed before the onset of expression, and *SIX3* was never switched on in a subset of the cultures. In either case, this points to an extended role for the node or its derivatives in promoting the expression of forebrain-specific genes. This likely includes a prolonged maintenance role for the axial mesendoderm, since *SIX3* is maintained for only 48 h in cultures with *CHORDIN* or prechordal plate tissue present (Fig. 4K,O,S,W). Similarly, *Gdf1*^−/−^ *Nodal*^+/−^ mouse embryos have impaired anterior axial mesendoderm formation, leading to a lack of *SIX3* expression and significant defects in the forebrain. These effects may stem from an excess of Wnt signalling, which is known to decrease *SIX3* expression (Lagutin et al., 2003). The node-derived prechordal mesendoderm likely provides signals that maintain forebrain identity, in part by providing Wnt antagonists (Foley et al., 1997; Rowan et al., 1999; Chapman et al., 2004; Fossat et al., 2015).

After the original organiser graft experiments by Spemann and Mangold (Spemann, 1921; Spemann and Mangold, 1924), a desire to find homologous structures, and the conflating of the results from graft experiments with normal development, has led to confusion surrounding the timing and mechanisms of neural induction (Martinez Arias and Steventon, 2018). Node graft assays have been useful for identifying the changes associated with neural induction and result in the development of a body axis (Dias and Schoenwolf, 1990; Storey et al., 1992; Trevers et al., 2018). However, they cannot necessarily be extrapolated to the role of the node in normal development. An emerging consensus view across vertebrates is that neural specification and regionalisation must occur earlier than morphological organiser development since ablation of the organiser (across vertebrate model organisms) does not significantly alter nervous system development and AP patterning (reviewed by Neaverson and Steventon, 2025). Using culture techniques with the neural tissue isolated from the organiser at progressively later stages has allowed us to pinpoint the periods of signal requirement for different aspects of neural development. This allowed us to definitively show that signals from the node and its precursors are important for the onset of *SOX2* in the neural plate, and subsequent posteriorisation of the neural plate through planar signals. These are then supported by vertical signals from the emerging axial mesendoderm, which continue to provide local patterning signals that maintain AP pattern and induce DV pattern in the overlying neural tissue.

## MATERIALS AND METHODS

### *Ex ovo* culture of chick embryos

Shaver brown chicken (*Gallus gallus domesticus*) eggs were obtained from MedEggs (Henry Stewart & Company, Norfolk, England). Eggs were incubated at 37.5°C in a humidified incubator within 1-2 days of arrival. Incubation timings to achieve the desired stage were guided by Hamburger and Hamilton (1992); for HH4, 18-22 h of incubation was necessary. Embryos were cultured using the EC culture method (Chapman et al., 2001). Embryos in culture were imaged and staged using the Leica MZ10F stereoscope with bright-field illumination, typically at 2.5× unless otherwise specified. For transgenic GFP embryos or grafts, a separate image was obtained of the GFP fluorescence, which was then combined with the bright-field image using FIJI, to produce a single composite image.

### Node and streak grafting

Transgenic cytoplasmic GFP eggs from the Roslin Institute (University of Edinburgh, Easter Bush, Scotland) (McGrew et al., 2008) were incubated at 37.5°C for 15-17 h to achieve HH3^+^ embryos to use as donors. Wild-type embryos incubated for the same duration were put into EC culture to be used as hosts. Donor GFP embryos were removed from the yolk and vitelline membrane, and transferred into PBS. The region to be grafted was cut out using a microdissection knife then aspirated into a 10 μl pipette and transferred to the host embryo culture. Using an eyelash knife, a small ‘pocket’ or hole was created in the mesendoderm layer at the outermost edge of the area pellucida. The piece of tissue was then pushed into the hole with the mesendoderm side facing down. Excess saline was then removed using a pipette, before incubation at 37.5°C for 24 h.

### Anterior embryo explant assay

Using HH2-HH6 embryos in EC culture, the primitive streak and posterior part of the embryo (including the associated area opaca tissue) were dissected out using a microdissection knife. Cuts were made slightly anterior to the node, then parallel to the primitive streak on either side, stopping just beyond the region corresponding to the prospective spinal cord (guided by fate maps in Fernández-Garre et al., 2002). For embryos at HH5-6, the cuts were made anterior to and around the visibly emerging axial mesendoderm. The segment was isolated by cutting diagonally across the embryo in a posterior-lateral direction, cutting through the area opaca, before removing the tissue with a pipette. Yolk and saline were removed from the embryo and the surrounding vitelline membrane. Then, a soldering iron set to 250°C was used to burn small regions of the vitelline membrane all around the embryo, creating a roughly circular barrier all around, to keep the anterior segment confined within the circle (Serrano Nájera et al., 2025).

### RNA extraction

HH3^+^ embryos were dissected from eggs into ice-cold PBS (Sigma-Aldrich). The primitive streak of each embryo was divided up into quarters, to give one node fragment (100% region), and one 75%, one 50% and one 25% region. 18 embryos were used in total, with corresponding streak sections pooled and transferred into 1 ml Trizol (ThermoFisher). Tissues were homogenised with a handheld tissue homogeniser, then 0.2 ml chloroform was added, before incubating at room temperature for 3 min. Samples were centrifuged for 15 min at 12,000 ***g*** at 4°C. The upper aqueous phase was transferred to a new tube, then 0.5 μl GlycoBlue and 0.5 ml isopropanol were added, before incubating for 10 min at room temperature, then centrifuging for 10 min at 12,000 ***g*** at 4°C. The supernatant was discarded and the pellet washed in 75% ethanol, then air dried at room temperature for 10 min. The pellet was then resuspended in nuclease-free water and incubated at 55°C for 15 min.

### RT-qPCR

Reverse transcription of RNA to cDNA was carried out using the Superscript III First-Strand Synthesis System (ThermoFisher) at 50°C for 45 min, before inactivating the reaction by heating to 70°C for 15 min. RNA was then removed using RNAse H digestion for 20 min at 37°C. qPCR was then performed using the QIAGEN Rotor-Gene PCR cycler and SYBR Green Mastermix, with reactions run in triplicate. Gene expression was quantified by standard curve analysis using a dilution series of cDNA from HH5 whole embryos. The Ct (threshold cycle) was calculated for each dilution in the series, using a threshold of 0.02, and used to create a log dilution-Ct graph for each gene. Using this, the relative concentration of each transcript in the unknown samples could be calculated. Dilutions were linearised and standardised relative to *ACTB*. Primer sequences can be found in Table S2.

### Embryo dissection, fixation and dehydration

Embryos were fixed by placing the embryo (from EC culture, attached to filter paper) in 4% PFA (paraformaldehyde, Sigma-Aldrich, 158127) in PBS, overnight at 4°C. The following day, embryos were removed from the vitelline membrane using sharp forceps and transferred to PBST (PBS+0.1% Tween). Embryos were then dehydrated using a series of 10 min graded methanol/PBST washes, starting with 2×PBST washes, then 25% methanol, 50% methanol and 75% methanol, then 100% methanol followed by storage at −20°C in 100% methanol.

### HCR RNA FISH

Gene expression was visualised using HCR RNA In Situ Hybridisation v3 (Choi et al., 2018). All buffers used for HCR were made in-house, according to the HCR RNA-FISH protocol (v3) for whole-mount chicken embryos. Embryos were first rehydrated using 10 min graded methanol/PBST washes in reverse order. The PBST was removed and replaced with a 10 μg/ml solution of proteinase K (Sigma, P4850) in PBST for 3 min at room temperature. This was replaced with 4% PFA for postfixing, for 20 min at room temperature, followed by two washes with 5×SSCT [SSC buffer (Thermo Scientific, J60839.K2)+0.1% Tween]. Embryos were then incubated for 30 min in hybridisation buffer at 37°C. HCR probes (obtained from Molecular Instruments, information provided in Table S3, with the exception of the NKX2.1 probes, which were generously provided by Kavitha Chinnaiya, University of Sheffield, UK) were diluted in hybridisation buffer to 4 pmol/ml and applied to the embryos for overnight incubation at 37°C. The next day, embryos were washed using four 20 min washes with probe wash buffer at 37°C, then two 5 min washes with SSCT. Embryos were then incubated in amplification buffer for 5 min. Fluorescent HCR hairpins (Molecular Instruments) were snap-cooled by incubation at 95°C for 90 s before cooling to room temperature. The hairpins were diluted to 60 pmol/ml in Amplification Buffer. Pre-amplification solution was removed and replaced with hairpin solution, followed by overnight incubation at room temperature in the dark. Hairpin solution was removed, and embryos were washed three times for 15 min each with SSCT. DAPI staining was performed by incubating embryos in DAPI (1:1000 in SSCT) for 30 min at room temperature. DAPI solution was removed and embryos were washed three times for 10 min each in SSCT.

### Confocal imaging

Fluorescently-stained embryos were mounted in VectaShield (Vector Laboratories, H-1000) between two glass coverslips (a smaller coverslip placed on top of a larger coverslip), separated by a layer of electrical tape. The edges of the small coverslip were sealed with nail polish. Confocal imaging was performed using the inverted Zeiss LSM700 microscope using either the 10× or 20× air objective.

### Image processing

Confocal images were processed in FIJI (Schindelin et al., 2012) to produce maximum intensity projections or summed slice projections. In some cases, individual images were stitched together using the ‘Pairwise Stitching’ function (Preibisch et al., 2009). Images of embryos containing both a bright-field and a GFP channel were overlaid using the ‘Merge Channels’ function. The area of *SOX2* expression in Fig. 1 was measured using the polygon tool and ‘Measure’ tool.

### Timelapse imaging

Live imaging of control embryos and anterior segments was performed using Nikon Eclipse Ti-E inverted wide-field microscope with a heated chamber (37°C), using a 10× air objective. Images were obtained at 45 min intervals. Movie files (.nd2) were processed in FIJI. For visualisation purposes, the forebrain, midbrain and hindbrain regions were manually tracked from the bright-field movies and overlaid on the timelapse recordings.

### Whole-mount imaging

Embryos were imaged using the Leica MZ10F stereoscope with brightfield illumination, typically at 2.5× unless otherwise specified. For embryos containing grafted regions, separate images were obtained of the GFP and bright-field channels, which were then combined in FIJI, as previously described.

### Statistical analysis

To assess differences in gene expression between conditions, a Fisher's Exact test was performed on 2×2 contingency tables comparing the number of embryos with positive and negative expression for each gene across conditions. Statistical analyses were carried out in Rstudio. A *P*-value threshold of 0.05 was used to determine statistical significance. Fisher's Exact test was selected, as it is appropriate for the small sample sizes and computes exact rather than approximated *P*-values.

## Supplementary Material



10.1242/develop.205685_sup1 Supplementary information
