# Supplementary material for: Dissecting planar and vertical organiser signals in early chick neural development
Source: Development. 2026 Jul 6;153(13):dev205685. doi: 10.1242/dev.205685 (PMC13380968; doi:10.1242/dev.205685)
Supplement: Supplementary file 1 [file develop-153-205685_review_history.pdf]

Dissecting planar and vertical organiser signals in early chick neural development

Alexandra Neaverson and Benjamin J. Steventon
DOI: 10.1242/dev.205685

Editor: James Briscoe

Review timeline

Original submission:	20 March 2026
Editorial decision:	2 May 2026
First revision received:	21 May 2026
Accepted:	3 June 2026

Original submission

First decision letter

MS ID#: dev.205685

MS TITLE: Dissecting planar and vertical organiser signals in early chick neural development

AUTHORS: Alexandra Neaverson and Benjamin J. Steventon

Dear Dr Steventon,

I have now received all the referees' reports on the above manuscript, and have reached a decision. The referees' comments are appended below.

As you will see, the referees find your anterior segment culture assay creative and your central question important, but identify several issues that must be addressed to support the conclusions. Most critically, you are asked to define the assay rigorously: clarify which tissues are included in the explants (particularly underlying endoderm and hypoblast), explain how the assay is modified at HH5 and HH6 given that axial mesoderm is already present, and characterise its reproducibility. I would particularly draw your attention to the comments of Reviewer 1. Please discuss the prior literature on planar neural induction in chick (Pera et al. 1999; Storey et al. 1995) and Xenopus (Sive et al. 1989; Elkouby et al. 2010) and sharpen your statement of novelty. The referees also request that you temper interpretations that cannot exclude alternative signal sources, clarify the basis for "posteriorisation" and Krox-20 induction and justify the claim that neural specification is complete by HH4.

If you are able to revise the manuscript along the lines suggested, which may involve further experiments, I will be happy to receive a revised version of the manuscript. Your revised paper will be re-reviewed by one or more of the original referees, and acceptance of your manuscript will depend on your addressing satisfactorily the reviewers' major concerns. Please also note that Development will normally permit only one round of major revision. If it would be helpful, you are welcome to contact us to discuss your revision in greater detail. Please send us a point-by-point response indicating your plans for addressing the referees' comments, and we will look over this and provide further guidance.

Please attend to all of the reviewers' comments and ensure that you upload both a 'clean' version of your Word file, along with a highlighted version clearly showing where you have made changes in

© 2026. Published by The Company of Biologists under the terms of the Creative Commons Attribution License (https://creativecommons.org/licenses/by/4.0/).

the revised manuscript. Please avoid using 'Tracked changes' in Word files as these are lost in PDF conversion. I should be grateful if you would also provide a point-by-point response detailing how you have dealt with the points raised by the reviewers in the 'Response to Reviewers' box. If you do not agree with any of their criticisms or suggestions please explain clearly why this is so.

Reviewer 1

SUMMARY OF THE ADVANCE MADE IN THIS PAPER AND ITS POTENTIAL SIGNIFICANCE TO THE FIELD

This paper addresses the interesting topic of how the neural cell state is established in the developing chick embryo. The authors use a "anterior segment culture assay" to investigate the role of the organiser region and emergent axial mesoderm in this process. The central claims of this paper are that planar signals from the early node are required for neural specification (Sox2 expression), that axial mesoderm contributes to stabilising anterior-posterior pattern and is required for dorsoventral patterning of the neural tube.

Some key papers are missing in the introductory review of the field and this is reflected in the interpretation of the data presented. A few examples are noted in suggestions for the authors. The "anterior segment culture assay" needs to be better defined and details of variations of this at different stages of development clarified. At present, this lack of clarity undermines confidence in the authors' interpretation of their experiments.

SUGGESTIONS TO AUTHORS

1) I am concerned about the authors "anterior segment culture assay". This appears to include underlying endoderm, rather than just a sheet of epiblast. If this is the case, this should be made clear in the main text. The underlying endoderm (which emerges from the early primitive streak) has been shown to be required to establish the prospective neural plate and defines the boundary between this region and the epidermis. This was shown by Pera et al 1999 (https://doi.org/10.1242/dev.126.1.63), where removal of the endoderm and associated hypoblast at HH3 (repeated at intervals thereafter to ensure no replacement by later emerging mesoderm) resulted in ectopic expression of epidermal markers e.g. Dlx5 and lack of neural plate markers including Sox2. These experiments should be cited and support a different interpretation of the anterior segment culture experiments reported here, where inclusion of emerging endoderm and hypoblast as well as planar signalling may underlie neural specification prior to emergence of the axial mesoderm from the node.

2) Evidence for the existence of planar signals within the epiblast / neural plate has been presented in previous work in the chick. The authors conclude from the anterior segments isolated at HH2-3 which increase Sox2/Sox3 expression with time to HH4 that "neural specification cues are therefore initially provided by planar signals from the PS". Ln 193-201. This was shown by Pera et al 1999 (https://doi.org/10.1242/dev.126.1.63), who placed neural plate grafts into the in area pellucida demonstrating planar transmission of signals that define the extent of the neural plate. A similar finding was also reported by Storey et al 1995 https://doi.org/10.1242/dev.121.2.417 who grafted epiblast from the node and just anterior to it to the area opaca. The overall conclusion from this paper was that the epiblast layer of node was a source of neural inducing signals.

3) The finding that "once neural identity is specified development can continue without additional planar neural inducing signals" ln 216-217 is based on HH4 anterior segments expressing Sox1 after 24h in the absence of axial mesoderm marker Chordin. This refers to planar signals not provided by the node/axial mesoderm, but there could be planar signals from within the neuroepithelium itself and in the anterior segment assay there could also be endoderm signals.

4) The experimental design for experiments presented in figure 2 requires further explanation. In this "The anterior segment culture method was used (fig 2A) separating the early neural plate from the primitive streak and posterior body at HH3+, HH4, HH5 or HH6 - using HH3+ as the youngest". Ln 225-227. This seems to indicate that anterior segment cultures at HH5 and 6 lack a node but these must still include axial mesoderm that has already emerged and is present below the neural plate (not shown in Fig 2A). If the authors dissected away this axial mesoderm this needs to be explained, but this would still complicate interpretation of these experiments as the

© 2026. Published by The Company of Biologists under the terms of the Creative Commons Attribution License (https://creativecommons.org/licenses/by/4.0/).

neural plate would have been exposed to vertical signals from this tissue. If the authors took a different more anterior region of "anterior segment" at these later stages this needs to be explained and findings interpreted accordingly.

5) There are some interesting findings presented here with respect to early timing of Sox gene expression.

6) The above concerns about the "anterior segment" assay undermine the authors' central conclusion that they provide evidence that "separate(s) the involvement of planar and vertical signals during early chick development, providing a timeline for the involvement of the developing organiser, the node proper, the node-derived axial mesendoderm for the development and maintenance of neural tissues in the chick embryo".

Reviewer 2

SUMMARY OF THE ADVANCE MADE IN THIS PAPER AND ITS POTENTIAL SIGNIFICANCE TO THE FIELD

The identity and source of signals that instruct neural induction, and regionalisation of neural tissues, is not resolved. Although various tissues and signals are known to play a role, uncoupling signals emanating from the node, and/or its derivatives such as prechordal plate mesoderm, or the primitive streak, has not been previously possible to determine. Additionally, to what extent the morphogenesis of the neural tube requires continuous contact with other tissues remains unclear. To begin to address these gaps, the authors have established a novel culture system in chick to isolate the prospective anterior neural plate from the node and primitive streak regions, enabling them to study the relative contributions from different tissues towards the process of neural induction and part of the process of regionalisation, that provides the anterior neural plate with more posterior character (hindbrain).

The authors demonstrate:
- a new technique that allows them to uncouple the neural inducing effects on the anterior neural plate, mediated at least in part by the organizer, versus its derivatives.
Using this approach, the key findings demonstrate that:
-the acquisition of a SOX2/3 identity can commence prior to formation of the node
-vertical signals from the underlying mesoderm are important for maintaining forebrain identity
-morphogenesis of the neural plate can occur in the absence of other midline tissues

The new technique developed here, which is creative and novel, represents an important contribution to the community to be able to pursue the molecular underpinnings of neural induction - a critically important topic at the heart of developmental biology.

SUGGESTIONS TO AUTHORS

I have some minor suggestions to help clarify the key messages and advances in the paper.

1. As the anterior segment culture represents a new technique, and has the capacity to be used further to dissect the molecular underpinnings of neural induction, it would be helpful to more clearly outline the reproducibility of the assay. Is the extent of neural induction (for example, relative domain size that is induced) relatively consistent across different cultures?

2. As the authors outline, the anterior segment culture represents a modification on previous techniques, to "ensure that all prospective neural plate regions were included" (see eg line 165-66). The authors also highlight that a "node graft can induce the entire spectrum of AP fates after grafting (Storey, Crossley and Stern, 1992; see also textbooks such as Gilbert and Barresi,2017)…". To what extent do these assays reported here relate to the generation of spinal cord, previously linked to the activation-transformation model? This is important to clarify in the text for readers outside of the neural induction field, particularly as "posterior" can be linked to different parts of the neuroaxis where distinct inductive mechanisms have been described.

3. On line 126, what is meant by "quickly posteriorize"? The precise mechanism responsible for the induction of Krox-20 positive cells is not clear in this assay, and is important for the

© 2026. Published by The Company of Biologists under the terms of the Creative Commons Attribution License (https://creativecommons.org/licenses/by/4.0/).

interpretation that the data presented (eg line 236-238) and further elaborated on in the discussion.

4. Could the authors clarify how they determine that acquisition of neural plate identity is completed by HH4 (see line 199-200)?

5. Model summary in Figure 5. It may be helpful to consider adding some references to the figure legend with regards to the key signals outlined, as these are not tested here.

Reviewer 3

SUMMARY OF THE ADVANCE MADE IN THIS PAPER AND ITS POTENTIAL SIGNIFICANCE TO THE FIELD

This is an interesting and elegant paper that advances the field. I have a few suggestions for textual change and one scientific question, but besides those points, the paper is excellent.

SUGGESTIONS TO AUTHORS

While the authors are generous citing chick sources and discussing them, less so with Xenopus. This can only strengthen their arguments, so it is worthwhile for them to add to expand the generality of the paper.

1. No citation (Xenopus) for the statements made on lines 115 -118 in the text. This should be addressed.

2. The seminal Sive et al. Paper in Cell from 1989 (Cell, Vol. 58, 171-180, July 14) is not cited and not even discussed. I think in light of your results, this paper needs to be addressed in both the introduction and discussion.

3. The authors mention the potential role non-axial mesoderm in neural AP patterning. There are studies in chick and Xenopus (Elkouby et al. Development 137, 1531-1541, 2010) addressing this issue and I think the implications need to be mentioned.

4. In terms of experimentation: Have the authors looked at any spinal cord markers in this assay? I think whether hindbrain was induced and spinal cord not, or both were induced in the explants, either way this would be an interesting observation and add even more impact to the paper.

First revision

Author response to reviewers' comments

We thank all three reviewers for their helpful comments that have improved the clarity of our manuscript, and to better contextualize our findings. Major changes are highlighted in yellow in response to these comments. The final word count is now: 7,649.

Reviewer 1: SUMMARY OF THE ADVANCE MADE IN THIS PAPER AND ITS POTENTIAL SIGNIFICANCE TO THE FIELD
This paper addresses the interesting topic of how the neural cell state is established in the developing chick embryo. The authors use a "anterior segment culture assay" to investigate the role of the organiser region and emergent axial mesoderm in this process. The central claims of this paper are that planar signals from the early node are required for neural specification (Sox2 expression), that axial mesoderm contributes to stabilising anterior-posterior pattern and is required for dorsoventral patterning of the neural tube.

Some key papers are missing in the introductory review of the field and this is reflected in the interpretation of the data presented. A few examples are noted in suggestions for the authors. The

© 2026. Published by The Company of Biologists under the terms of the Creative Commons Attribution License (https://creativecommons.org/licenses/by/4.0/).

"anterior segment culture assay" needs to be better defined and details of variations of this at different stages of development clarified. At present, this lack of clarity undermines confidence in the authors' interpretation of their experiments.

We thank this reviewer for pointing out some key aspects of our experiments that were not previously clear. These have now been clarified in response to the following specific points:

SUGGESTIONS TO AUTHORS

1) I am concerned about the authors "anterior segment culture assay". This appears to include underlying endoderm, rather than just a sheet of epiblast. **If this is the case, this should be made clear in the main text.** The underlying endoderm (which emerges from the early primitive streak) has been shown to be required to establish the prospective neural plate and defines the boundary between this region and the epidermis. This was shown by Pera et al 1999 (https://doi.org/10.1242/dev.126.1.63), where removal of the endoderm and associated hypoblast at HH3 (repeated at intervals thereafter to ensure no replacement by later emerging mesoderm) resulted in ectopic expression of epidermal markers e.g. Dlx5 and lack of neural plate markers including Sox2. **These experiments should be cited and support a different interpretation of the anterior segment culture experiments reported here, where inclusion of emerging endoderm and hypoblast as well as planar signalling may underlie neural specification prior to emergence of the axial mesoderm from the node.**

We thank the reviewer for highlighting this paper, that we have now cited extensively in the revised manuscript. Our intention was to dissect the role of organiser signals in neural induction within the epiblast that has been primed for neural induction by these signals, therefore it was essential to retain hypoblast within our anterior segment cultures. We have now made it clear that this work aims to dissect the role of planar and vertical signals derived from the node- in the context of the neural plate having already received hypoblast signals.

These changes are highlighted in yellow in the revised manuscript and are listed here:

1. The role of the hypoblast and early mesendoderm is now highlighted in the abstract.
2. Pera et al (1999) has been added to the introduction.
3. In Results section 1, we have added clarification that the mesendodermal layer was left intact in anterior segment cultures.
4. Concluding statements in Results section 1 have been adapted to highlight the permissive role of vertical mesendoderm-derived signals.
5. An additional statement has been added to the discussion to highlight that organiser-derived signals work in conjunction with signals from the early endoderm
6. In any instance where planar and vertical signals are mentioned, the tissue that we are referring to as the source of the signal has been clarified.

2) Evidence for the existence of planar signals within the epiblast / neural plate has been presented in previous work in the chick. The authors conclude from the anterior segments isolated at HH2-3 which increase Sox2/Sox3 expression with time to HH4 that "neural specification cues are therefore initially provided by planar signals from the PS". Ln 193-201. This was shown by Pera et al 1999 (https://doi.org/10.1242/dev.126.1.63), who placed neural plate grafts into the in area pellucida demonstrating planar transmission of signals that define the extent of the neural plate. A similar finding was also reported by Storey et al 1995 https://doi.org/10.1242/dev.121.2.417 who grafted epiblast from the node and just anterior to it to the area opaca. The overall conclusion from this paper was that the epiblast layer of node was a source of neural inducing signals.

We do not claim that this work provides the first evidence of planar signalling in neural induction, but rather use our anterior segment culture assay to determine the relative order of these organiser-derived inductive signals. We have modified the following sections of the manuscript to make this clear:

1. Contributions of Pera et al 1999 and Storey et al 1995 have been acknowledged in the introduction.
2. The final paragraph of the introduction has been modified to better explain the aims and conclusions of this work: “Here we employ an anterior tissue isolation method to determine

© 2026. Published by The Company of Biologists under the terms of the Creative Commons Attribution License (https://creativecommons.org/licenses/by/4.0/).

the relative contributions of planar signals from the organiser and vertical signals from its derivatives, and the timing that these signals are required, for neural specification and anterior-posterior patterning."

3) The finding that "once neural identity is specified development can continue without additional planar neural inducing signals" ln 216-217 is based on HH4 anterior segments expressing Sox1 after 24h in the absence of axial mesoderm marker Chordin. This refers to planar signals not provided by the node/axial mesoderm, but there could be planar signals from within the neuroepithelium itself and in the anterior segment assay there could also be endoderm signals.

Our focus here is to dissect planar vs. vertical signals from the organiser (rather than all potential sources of planar and vertical signals), and agree with the reviewer that this was not clear in the previous version of the manuscript. We have now modified the wording of the manuscript throughout to make this clear.

4) The experimental design for experiments presented in figure 2 requires further explanation. In this "The anterior segment culture method was used (fig 2A) separating the early neural plate from the primitive streak and posterior body at HH3+, HH4, HH5 or HH6 - using HH3+ as the youngest". Ln 225-227. This seems to indicate that anterior segment cultures at HH5 and 6 lack a node but these must still include axial mesoderm that has already emerged and is present below the neural plate (not shown in Fig 2A). If the authors dissected away this axial mesoderm this needs to be explained, but this would still complicate interpretation of these experiments as the neural plate would have been exposed to vertical signals from this tissue. If the authors took a different more anterior region of "anterior segment" at these later stages this needs to be explained and findings interpreted accordingly.

The cutting method was changed for HH5 and HH6 embryos to exclude the axial mesendoderm. Therefore, we have excluded the vertical signals from the axial mesendoderm in anterior segments isolated at HH4 or younger, before this tissue has emerged. We fully acknowledge that HH5 or HH6 neural plates would already have been exposed to vertical signals from the axial mesendoderm, but do not draw conclusions as to whether vertical or planar signals are required as cells initially emerge from the node.

We have made the following changes to clarify this point:

1. Explanation of this has been added to Results section 2.
2. Explanation of this has been added to the Materials and Methods.
3. Wording changed to emphasise that we make conclusions about planar signals using the data from HH4 anterior segment cultures:
   "Since 62% of HH4 cultures were able to express KROX-20 following 24h culture, this indicates that AP pattern acquisition, at least up to hindbrain level, primarily occurs through cues from planar, node-derived signals between $HH3^{+}$ and HH4."
4. Wording has been changed in the concluding statement to emphasise that the hindbrain specification does occur prior to HH4, but that additional signals post-HH4 do play a role here:
   "Overall, this indicates that AP patterning occurs in a stepwise fashion and is predominantly orchestrated by planar node-derived signals, but hindbrain fates take longer to fully establish, and may be supported by additional caudalising signals from axial and non-axial mesoderm"
5. Figure 2 has been modified to show the method for cutting post-HH4 anterior segments.

5) There are some interesting findings presented here with respect to early timing of Sox gene expression.

6) The above concerns about the "anterior segment" assay undermine the authors' central conclusion that they provide evidence that "separate(s) the involvement of planar and vertical signals during early chick development, providing a timeline for the involvement of the developing organiser, the node proper, the node-derived axial mesendoderm for the development and maintenance of neural tissues in the chick embryo".

© 2026. Published by The Company of Biologists under the terms of the Creative Commons Attribution License (https://creativecommons.org/licenses/by/4.0/).

The previous changes have helped to clarify that we only aim to separate signals from the organiser (planar) with signals from the axial mesendoderm, as opposed to dissecting all of the contributing signals in neural specification.

Reviewer 2: SUMMARY OF THE ADVANCE MADE IN THIS PAPER AND ITS POTENTIAL SIGNIFICANCE TO THE FIELD

The identity and source of signals that instruct neural induction, and regionalisation of neural tissues, is not resolved. Although various tissues and signals are known to play a role, uncoupling signals emanating from the node, and/or its derivatives such as prechordal plate mesoderm, or the primitive streak, has not been previously possible to determine. Additionally, to what extent the morphogenesis of the neural tube requires continuous contact with other tissues remains unclear. To begin to address these gaps, the authors have established a novel culture system in chick to isolate the prospective anterior neural plate from the node and primitive streak regions, enabling them to study the relative contributions from different tissues towards the process of neural induction and part of the process of regionalisation, that provides the anterior neural plate with more posterior character (hindbrain).

The authors demonstrate:
- a new technique that allows them to uncouple the neural inducing effects on the anterior neural plate, mediated at least in part by the organizer, versus its derivatives.
Using this approach, the key findings demonstrate that:
-the acquisition of a SOX2/3 identity can commence prior to formation of the node
-vertical signals from the underlying mesoderm are important for maintaining forebrain identity
-morphogenesis of the neural plate can occur in the absence of other midline tissues

The new technique developed here, which is creative and novel, represents an important contribution to the community to be able to pursue the molecular underpinnings of neural induction - a critically important topic at the heart of developmental biology.

We thank the reviewer for these comments, that we feel provide a succinct summary of our major findings.

SUGGESTIONS TO AUTHORS

I have some minor suggestions to help clarify the key messages and advances in the paper.

1. As the anterior segment culture represents a new technique, and has the capacity to be used further to dissect the molecular underpinnings of neural induction, it would be helpful to more clearly outline the reproducibility of the assay. Is the extent of neural induction (for example, relative domain size that is induced) relatively consistent across different cultures?

We have gone back to our images to quantify the size and number of expression domains, as summarised in the table below. As expected, domain size is highly variable between individual embryos, whose overall embryo size is also variable. Unfortunately, we don't have whole embryo images for each experiment to normalise expression domain size to embryo size. However, we have now included our counts of expression domain numbers in a revised version of the manuscript.

<u>SOX3</u>

STAGE	Mean number of domains	Mean area per domain (μm2)	Std Dev
HH2	1	127414.636	na

© 2026. Published by The Company of Biologists under the terms of the Creative Commons Attribution License (https://creativecommons.org/licenses/by/4.0/).

HH3	1	665198.639	329195.5065
HH3+	1	56852.7405	21901.2643
HH4	1.25	117377.631	88050.68987

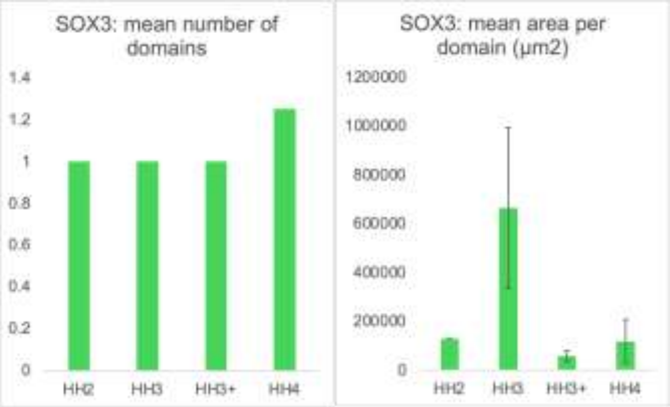

SOX2

STAGE	Mean number of domains	Mean area per domain (μm2)	Std Dev
HH2	1	154220.734	na
HH3	1	444391.262	360899.9423
HH3+	1.5	66512.2817	32255.35858
HH4	1.25	119125.299	99762.52937

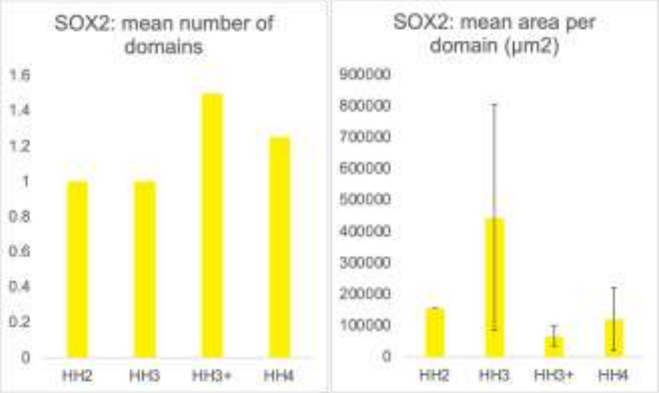

OTX2

© 2026. Published by The Company of Biologists under the terms of the Creative Commons Attribution License (https://creativecommons.org/licenses/by/4.0/).

Stage	Mean number of domains	Mean area per domain (µm2)	SD
HH3+	3	61305.15775	16796.00765
HH4	2.666666667	98049.0644	101028.3917
HH5	3	89574.80231	41237.31832

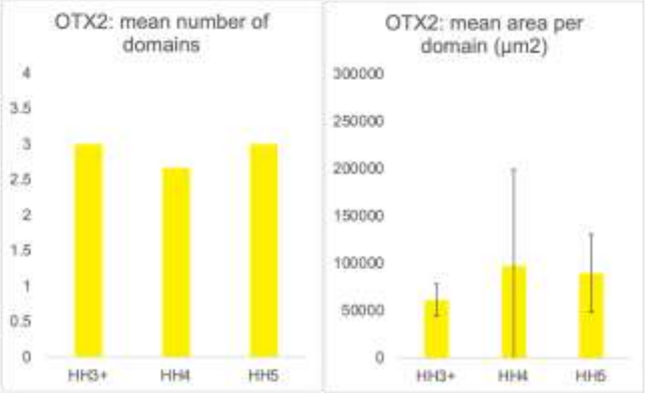

KROX-20

Stage	Mean number of domains	Mean area per domain (µm2)	SD
HH4	0.916666667	24641.50294	28125.27969
HH5	1.416666667	13269.70417	5353.604454
HH6	2.2	11105.63856	7760.803742

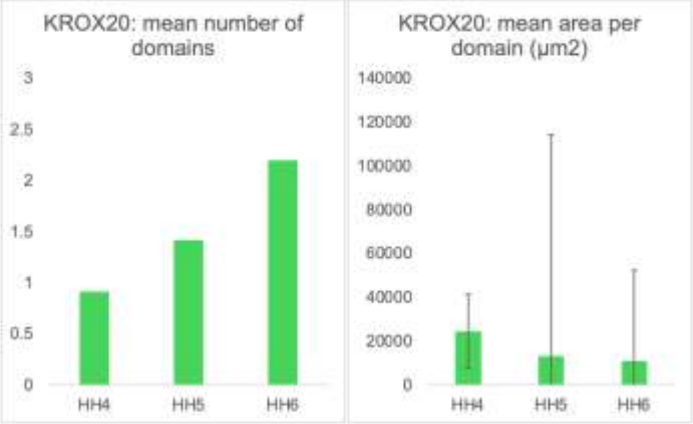

Summary of changes made:

© 2026. Published by The Company of Biologists under the terms of the Creative Commons Attribution License (https://creativecommons.org/licenses/by/4.0/).

We have decided to include the domain number measurements for SOX2, SOX3, OTX2 and KROX-20. We believe the increase in KROX-20 domains over time may be meaningful.

In the results:

- In the majority of *SOX2/SOX3*$^+$ cultures, a single expression domain developed, but in two samples (from HH3$^+$ and HH4 embryos) two separate domains emerged, likely a consequence of excising the primitive streak affecting the geometry of the early neural plate.
- The number of OTX2$^+$ expression domains was variable, with all cultures having between 2 and 4 separate OTX2$^+$ regions, and this was independent of the stage isolated. In contrast, the mean number of *KROX-20*$^+$ domains increased with isolation stage (HH4 – 0.92 domains; HH5 – 1.42 domains; HH6 – 2.2 domains), with resemblance to a normal embryo (Figure 2C) increasing with stage.

2. As the authors outline, the anterior segment culture represents a modification on previous techniques, to "ensure that all prospective neural plate regions were included" (see eg line 165-66). The authors also highlight that a "node graft can induce the entire spectrum of AP fates after grafting (Storey, Crossley and Stern, 1992; see also textbooks such as Gilbert and Barresi,2017)…". To what extent do these assays reported here relate to the generation of spinal cord, previously linked to the activation-transformation model? This is important to clarify in the text for readers outside of the neural induction field, particularly as "posterior" can be linked to different parts of the neuroaxis where distinct inductive mechanisms have been described.

We agree with the reviewer that this is an important point, however we are not able to provide a complete answer to this question. We have observed that anterior segments express more posterior spinal cord markers (Hoxb8), however it was accompanied by Tbx6 expression, so does not allow for us to exclude the idea that NMPs might be present. Nevertheless, we have modified the manuscript to consider this point:

Complete changes:

1. We have changed the wording to "all regions of the prospective brain along the AP axis were included", to be clear that we were looking for fates up to the level of the hindbrain.
2. Clarification that AP pattern acquisition ***up to hindbrain level*** occurs through cues from planar, node-derived signals between HH3$^+$ and HH4 and via Activation-Transformation.
3. Replacement of 'posterior' with 'hindbrain' towards the end of the second results section.
4. Addition to the discussion, paragraph 5 "It remains to be seen whether spinal cord specification occurs in these cultures; there is ongoing debate about whether any part of the spinal cord arises through Activation-Transformation (Nieuwkoop, 1952)), or from bipotent neuromesodermal progenitors (NMps) (Cambray and Wilson, 2002, 2007; Tzouanacou *et al.*, 2009; Wymeersch *et al.*, 2016). Such an experiment would require precise co-staining for neural and mesodermal markers alongside lineage analysis in order to rule out the presence of NMps."

3. On line 126, what is meant by "quickly posteriorize"? The precise mechanism responsible for the induction of Krox-20 positive cells is not clear in this assay, and is important for the interpretation that the data presented (eg line 236-238) and further elaborated on in the discussion.

What we are referring to here is that at HH3+, none of the cultures are able to express Krox-20, then at HH4, a substantial number (62%) of cultures have been transformed and can now express Krox-20. This means that the posteriorisation of the prospective brain, i.e. specification of the hindbrain, must happen quite quickly in between HH3+ and HH4. However, we agree that 'quickly posteriorize' is misleading in that it suggests that we are proposing a specific mechanism, which we did not intend.

Completed changes:

This sentence has been modified: "We find that neural specification is a gradual process that requires planar signals from the anterior primitive streak, and that subsequent planar signals from the node also promote hindbrain specification."

4. Could the authors clarify how they determine that acquisition of neural plate identity is completed by HH4 (see line 199-200)?

© 2026. Published by The Company of Biologists under the terms of the Creative Commons Attribution License (https://creativecommons.org/licenses/by/4.0/).

We determine that neural plate identity has been acquired by HH4 through demonstrating that SOX2 is expressed in 100% of anterior segment cultures, even in the absence of CHORDIN. Since SOX2 is generally considered to be the earliest marker of the definitive neural plate, this suggests that the presence of CHORDIN-positive node/axial mesoderm is no longer required for SOX2 to be stably expressed in the neural plate.

Complete changes:

- wording has been changed: This suggests that neural specification is a gradual process, rather than a switch occurring at a defined stage, and that the acquisition of neural plate identity is initiated prior to node formation. However, by HH4, it does not require further neural specification signals from the node.

5. Model summary in Figure 5. It may be helpful to consider adding some references to the figure legend with regards to the key signals outlined, as these are not tested here.

We thank the reviewer for this suggestion and have now added citations to the Figure 5 legend.

Reviewer 3: SUMMARY OF THE ADVANCE MADE IN THIS PAPER AND ITS POTENTIAL SIGNIFICANCE TO THE FIELD

This is an interesting and elegant paper that advances the field. I have a few suggestions for textual change and one scientific question, but besides those points, the paper is excellent.

SUGGESTIONS TO AUTHORS

While the authors are generous citing chick sources and discussing them, less so with Xenopus. This can only strengthen their arguments, so it is worthwhile for them to add to expand the generality of the paper.

1. No citation (Xenopus) for the statements made on lines 115 -118 in the text. This should be addressed.

We agree with the reviewer on this point and have now acknowledged several important Xenopus papers about AP patterning via planar signals and Activation-Transformation.
Completed changes:
Added to the introduction:
Studies in *Xenopus* have shown that planar signals from the dorsal mesoderm are key for AP pattern acquisition in the neurectoderm (Altaba, 1992; Doniach, Phillips and Gerhart, 1992) and that AP patterning occurs progressively, with more posterior brain regions passing through a temporary anterior state until they are exposed to later signals from the dorsal mesoderm (Durston *et al.*, 1989; Sive, Hattori and Weintraub, 1989; Cox and Hemmati-Brivanlou, 1995). However, others argue that vertical signals from early involuting mesoderm are the primary signal in *Xenopus* AP patterning (Chen *et al.*, 2000), or that vertical signals are necessary for AP pattern refinement (Poznanski and Keller, 1997).

2. The seminal Sive et al. Paper in Cell from 1989 (Cell, Vol. 58, 171-180, July 14) is not cited and not even discussed. I think in light of your results, this paper needs to be addressed in both the introduction and discussion.

Thank you for pointing to this paper. We have acknowledged the findings of this paper in both the introduction (see above) and the discussion.
Completed changes:
Discussion - sentence amended: These results give evidence for Nieuwkoop's Activation-Transformation model of neural development (Nieuwkoop, 1952) and resemble those of the experiments carried out by (Sive, Hattori and Weintraub, 1989) in *Xenopus*, who demonstrated that cells initially specified as the far-anterior cement gland tissue are transformed into more posterior fates after prolonged contact with dorsal mesoderm.

© 2026. Published by The Company of Biologists under the terms of the Creative Commons Attribution License (https://creativecommons.org/licenses/by/4.0/).

3. The authors mention the potential role non-axial mesoderm in neural AP patterning. There are studies in chick and Xenopus (Elkouby et al. Development 137, 1531-1541, 2010) addressing this issue and I think the implications need to be mentioned.

We have added additional text in the discussion to reflect that additional contact with paraxial mesoderm-derived signals may influence hindbrain patterning in later stage (HH5-6) cultures. The paraxial mesoderm is an additional source of signals, including Wnt, that is known to promote posterior neural fates in chick, zebrafish and *Xenopus* (Muhr, Jessell and Edlund, 1997; Woo and Fraser, 1997; Muhr *et al.*, 1999; Elkouby *et al.*, 2010). Prolonged contact with these signals post-HH4 may be the reason why KROX-20 domains appear more frequently and more refined in cultures isolated at HH5-6.

4. In terms of experimentation: Have the authors looked at any spinal cord markers in this assay? I think whether hindbrain was induced and spinal cord not, or both were induced in the explants, either way this would be an interesting observation and add even more impact to the paper.
This has been addressed in response to a similar comment from reviewer 2. We do have evidence that anterior segment cultures express more posterior spinal cord markers such as Hoxb8, but this is complicated by the accompanied expression of Tbx6, marking paraxial mesoderm progenitors. The presence of Tbx6 does not allow us to make any robust conclusions about whether spinal cord tissue is specified via planar signals/activation-transformation, as bi-fated NMPs may be present. For this reason we have decided to exclude it from this paper, however we have discussed this point further in a revised discussion.

Second decision letter

MS ID#: dev.205685R1

MS TITLE: Dissecting planar and vertical organiser signals in early chick neural development

AUTHORS: Alexandra Neaverson and Benjamin J. Steventon

Dear Dr Steventon,

I am happy to tell you that your manuscript has been accepted for publication in Development, pending our standard publication integrity checks. It was accepted on 03 Jun 2026. You will see that Referee 1 has a suggestion for a slight change in wording. I will leave it up to you whether you think this is helpful

Reviewer 1

SUGGESTIONS TO AUTHORS

The authors have reasonably addressed this reviewer's concerns in the revised manuscript. The Introduction now better captures the findings of previous studies localising the tissue origins of signals involved in neural specification. The aims of this new study and the central anterior segment culture assay have been clarified; in particular the exclusion of axial mesoderm that has emerged in later stage anterior explants is now noted and indicated in the revised figure 2. Overall this paper makes a useful contribution to our understanding of how neural cell fate is acquired.

A further thought for the authors to consider is the use of the term segment in the "anterior segment culture assay" - given that segments and segmentation refer to a completely other topic and this terminology may affect key word search etc, could this be just "anterior explant assay" or "anterior embryo explant assay" ?

Reviewer 2

© 2026. Published by The Company of Biologists under the terms of the Creative Commons Attribution License (https://creativecommons.org/licenses/by/4.0/).

SUMMARY OF THE ADVANCE MADE IN THIS PAPER AND ITS POTENTIAL SIGNIFICANCE TO THE FIELD

The main advance of the paper details a new technique that allows the authors to uncouple the neural inducing effects on the anterior neural plate, mediated at least in part by the organizer, versus its derivatives.
Using this approach the following is shown:
-the acquisition of a SOX2/3 identity can commence prior to formation of the node
-vertical signals from the underlying mesoderm are important for maintaining forebrain identity
-morphogenesis of the neural plate can occur in the absence of other midline tissues

The new technique developed here, which is creative and novel, represents an important contribution to the community to be able to pursue the molecular underpinnings of neural induction - a critically important topic at the heart of developmental biology.

SUGGESTIONS TO AUTHORS

Thank you to the authors for making the revisions to the text and including the new datasets. I have no further suggestions.

© 2026. Published by The Company of Biologists under the terms of the Creative Commons Attribution License (https://creativecommons.org/licenses/by/4.0/).
